# Chemical attribution of the homemade explosive ETN - Part II: Isotope ratio mass spectrometry analysis of ETN and its precursors

**DOI:** 10.1016/j.forsciint.2020.110344

**Published:** 2020-08

**Authors:** Karlijn Bezemer, Lindsay McLennan, Rosanne Hessels, Jorien Schoorl, Jos van den Elshout, Antoine van der Heijden, Annemieke Hulsbergen, Mattijs Koeberg, Taylor Busby, Alexander Yevdokimov, Eva de Rijke, Peter Schoenmakers, James Smith, Jimmie Oxley, Arian van Asten

**Affiliations:** a Van ‘t Hoff Institute for Molecular Sciences, Faculty of Science, University of Amsterdam, the Netherlands; b Netherlands Forensic Institute, The Hague, the Netherlands; c University of Rhode Island, Department of Chemistry, Kingston, RI, USA; d Institute for Biodiversity and Ecosystem Dynamics, Faculty of Science, University of Amsterdam, the Netherlands; e Dept. Energetic Materials, TNO Technical Sciences, Den Haag, the Netherlands; f CLHC, Amsterdam Center for Forensic Science and Medicine, University of Amsterdam, P.O. Box 94157, 1090 GD Amsterdam, the Netherlands

**Keywords:** Erythritol tetranitrate (ETN), Explosives, Homemade explosive, Isotope ratio mass spectrometry (IRMS), Chemical profiling, Attribution, Precursors, Forensic science

## Abstract

In this follow-up study the collaboration between two research groups from the USA and the Netherlands was continued to expand the framework of chemical attribution for the homemade explosive erythritol tetranitrate (ETN). Isotope ratio mass spectrometry (IRMS) analysis was performed to predict possible links between ETN samples and its precursors. Carbon, nitrogen, hydrogen and oxygen isotope ratios were determined for a wide variety of precursor sources and for ETN samples that were prepared with selected precursors. The stability of isotope ratios of ETN has been demonstrated for melt-cast samples and two-year old samples, which enables sample comparison of ETN in forensic casework independent of age and appearance. Erythritol and nitric acid (or nitrate salt) are the exclusive donor of carbon and nitrogen atoms in ETN, respectively, and robust linear relationships between precursor and the end-product were observed for these isotopes. This allowed for defining isotopic enrichment ranges for carbon and nitrogen that support the hypothesis that a given erythritol or nitrate precursor was used to synthesize a specific ETN batch. The hydrogen and oxygen atoms in ETN do not originate from one exclusive donor material, making linkage prediction more difficult. However, the large negative enrichments observed for both isotopes do provide powerful information to exclude suspected precursor materials as donor of ETN. Additionally, combing the isotopic data of all elements results in a higher discrimination power for ETN samples and its precursor materials. Combining the findings of our previously reported LC–MS analysis of ETN with this IRMS study is expected to increase the robustness of the forensic comparison even further. The partially nitrated impurities can provide insight on the synthesis conditions while the isotope data contain information on the raw materials used for the production of ETN.

## Highlights

• Follow-up of the unique study on the explosive ETN by two research groups.
• ETN samples were synthesized using a wide variety of selected precursor sources.
• IRMS analysis of ETN and its precursors for chemical attribution.
• Isotope ratios allow for ETN sample comparison independent of age and appearance.
• IRMS data is useful to investigate which raw materials were used for ETN synthesis.

## 1 Introduction

Erythritol tetranitrate (ETN) is a nitrate ester homemade explosive (HME) that is gaining popularity among amateurs, criminals, and terrorist bomb makers. The recent increase in accessibility of the precursor material erythritol as a low-calorie sugar substitute, and synthesis procedures that can relatively easily be performed in a homemade setting, have made ETN attractive for its use in improvised explosive devices (IEDs) [1,2]. In addition, ETN is less susceptible to accidental detonation compared to the frequently used and popular homemade explosive triacetone triperoxide (TATP). The explosive TATP has been used in numerous incidents, including the London subway attacks (2005), the Paris suicide bombers (2015), the Brussels Bombings (2016), and the Manchester Arena Bombing (2017). However, accidental detonation of TATP, which occurred in the Barcelona bomb plot in 2017, might persuade criminals and terrorist groups to use alternative HMEs (such as ETN) in future attacks. This makes it forensically relevant to detect and identify ETN to reveal potential explosive threats. Additionally, it is important to focus forensic explosives investigations on providing detailed information on production and origin of the explosive ETN and its precursors. This might give valuable leads to suspects or production sites involved, which can ultimately help to eliminate an explosive threat.

We have previously reported a study where two large sample sets of ETN were synthesized independently by two forensic laboratories using different synthesis routes and precursors [3]. The synthesis of ETN was performed by nitration of erythritol using either nitric acid (mixed acid route) or a nitrate salt (nitrate salt route). Both synthesis routes are likely to be encountered in homemade settings, because their precursors are relatively easy to acquire and there is no need for specialized laboratory equipment. The previous study showed limited options for impurity profiling using liquid chromatography-mass spectrometry (LC–MS) methods. No raw material predictions could be made from the analysis of partially nitrated erythritol by-products and additional erythritol impurities due the high food grade quality of the erythritol precursors.

Besides the analysis of impurities and by-products for chemical profiling of explosives, stable isotope analysis can be used to differentiate otherwise chemically identical materials. Several studies have focused on isotope analysis for forensic comparison of explosives sources, and to provide information on which precursors could have been used to synthesize explosive materials. Benson et al. [4,5] demonstrated the use of isotope ratio mass spectrometry (IRMS) to differentiate between explosives sources of ammonium nitrate (AN), pentaerythritol tetranitrate (PETN) and TATP. Brust et al. [6] combined nitrogen and oxygen isotope compositions with elemental analysis to obtain a higher discrimination power for AN samples. Other studies have increased the potential to differentiate between AN sources by separate analysis of the nitrogen isotopic composition of the ammonium and nitrate ions [7,8]. A study of Howa et al. [9] focused on the carbon and nitrogen isotope compositions of factory produced RDX and HMX explosives. Differentiation of C4-type plastic explosives was studied by analyzing the isotopic compositions of the isolated components (binder, plasticizer) [10]. Lock et al. [11,12] showed the potential for linkage of the explosives RDX and hexamethylene triperoxide diamine (HMTD) to its precursor material hexamine based on the carbon and nitrogen isotope ratios. In a previous study of our group [13], various sources of the precursor materials acetone and hydrogen peroxide were used to synthesize TATP samples and to study the isotopic fractionations. Bulk IRMS and compound specific gas chromatography (GC)-IRMS demonstrated the differentiation of TATP samples and the linkage to its acetone precursor based on carbon and hydrogen isotope ratios. The forensic application of isotope analysis for differentiation of urea nitrate (UN) was demonstrated by Aranda et al. [14], who also showed that the carbon and nitrogen isotope values can be used to potentially link urea or nitric acid precursors to corresponding UN samples. A similar study by Howa et al. [15] indicated that the carbon isotopic composition of PETN is dependent on its pentaerythritol (PE) precursor isotopic value, whereas the nitrogen isotope ratio of PETN is influenced by the nitric acid used for the nitration of PE.

A similar isotopic relationship might prove forensically useful for the explosive ETN and its erythritol and nitrate precursors. Therefore, this paper builds on our previously reported study, which focused on impurity analysis of partially nitrated erythritol using LC–MS analysis, by expanding the framework of chemical attribution for forensic explosives intelligence using IRMS to analyze the isotopic compositions of ETN and its precursors. The main objective was to investigate whether ETN samples can be differentiated based on their isotope ratios and to study linkage of ETN samples to their erythritol and nitrate starting materials. The unique collaboration between two independent forensic laboratories provided ETN samples that were synthesized with a wide variety of different precursor materials and synthesis conditions to study the effects on the final isotope ratios in the ETN samples. The previously synthesized ETN samples were included in this study to investigate potential degradation effects. Expanding the framework of chemical attribution with IRMS analysis could ultimately allow for predictions in forensic explosives casework concerning precursor materials that could have been used in the homemade synthesis of ETN. Such information can provide useful tactical leads that ultimately could result in the arrest of perpetrators and the prevention of successful attacks involving ETN.

## 2 Experimental

### 2.1 Precursor materials

The erythritol samples analyzed in this study originated from various sources. Different brands of pure erythritol and mixtures of erythritol with other artificial sweeteners were acquired in the USA and in the Netherlands. In total 39 samples were analyzed divided in five different erythritol categories: 100% erythritol (20 samples), 85% erythritol (1 sample), erythritol with stevia (10 samples), brown sugar erythritol (4 samples), and erythritol mixtures (4 samples). A detailed overview of the erythritol sample collection is listed in Table S1 of the supplementary information, including information on brand, composition, country and year of purchase, production or distribution country, and batch number. All erythritol samples were analyzed with IRMS to determine their hydrogen (δ^2^H), carbon (δ^13^C) and oxygen (δ^18^O) isotope ratios.

The nitrogen (δ^15^N) and δ^18^O isotope ratios of ten potassium nitrate (KNO_3_ 1–10) samples were determined. As alternative nitrate sources, an ammonium nitrate (NH_4_NO_3_) and a sodium nitrate (NaNO_3_) sample were added to the nitrate salt precursor selection. Fuming nitric acid, used for nitration of erythritol in the mixed acid synthesis route, was purchased from Millipore Sigma in the USA and from Merck in the Netherlands. Samples of nitric acid were converted to KNO_3_ prior to isotope ratio mass spectrometry (IRMS) analysis *via* modified literature methods [14,16]. A 2.0 mL aliquot of nitric acid was diluted in 11.0 mL of deionized water. The acid was neutralized with 1 M equivalent of potassium hydroxide as checked with pH paper. The water was evaporated, leaving behind a white solid. This white solid was washed with multiple aliquots of methanol to remove excess potassium hydroxide. The solid KNO_3_ product was dried at 60 °C overnight and stored in a desiccator until use. In this study four nitric acid samples were analyzed (HNO_3_ 1–4). Detailed information on the nitrate precursor sample collection is listed in Table S2 of the supplementary information, including sample information on brand, purity, and batch numbers.

### 2.2 ETN synthesis

*Warning:* Erythritol tetranitrate (ETN) is a powerful explosive, similar to pentaerythritol tetranitrate (PETN), which is considered a primary explosive under some circumstances. For forensic scientific purposes, like all explosives, ETN should only be synthesized and handled in small quantities and by authorized and qualified personnel.

In 2017, synthesis of ETN was performed at two locations: University of Rhode Island (URI) in the USA and TNO Technical Sciences in the Netherlands, which resulted in two sample sets: ETN-USA and ETN-NL, respectively. Samples were produced following the mixed acid and nitrate salt synthesis routes and details on the ETN samples synthesized in 2017 were recently reported in our Part I study [3]. In this follow-up study, the 34 ETN samples that were previously synthesized using standard synthesis conditions with different erythritol precursors (samples USA-MA1-6, USA-NS1-6, NL-MA1-10 and NL-NS 1-10) were analyzed using IRMS. The standard synthesis conditions included the addition of 9.3 mL of nitric acid to an erythritol/sulfuric acid mixture (mixed acid route) or the addition of 10 g potassium nitrate in sulfuric acid (nitrate salt route). In both routes, ETN was allowed to form in the mixtures for one hour at room temperature. In addition to the use of potassium nitrate, two samples were prepared using alternative nitrate salts: sodium nitrate (sample NL-NS11) and ammonium nitrate (sample NL-NS12). Another 17 ETN samples (that were prepared in 2017 with standard erythritol precursors) were included in this study to investigate the effects of different synthesis conditions on isotope fractionation. These different synthesis conditions included varying reaction times (samples USA-MA8-10, USA-NS7-10 and NL-MA14), replacing lab-grade sulfuric acid with battery acid (samples NL-MA13 and NL-NS14) and varying washing and recrystallization solutions (samples NL-MA19-21 and NL-NS19-21). For the ETN-USA samples the δ^13^C and δ^15^N isotope values were determined using IRMS analysis performed at URI (USA), and for the ETN-NL samples the δ^13^C, δ^15^N, δ^2^H and δ^18^O isotope ratios were analyzed at the Institute for Biodiversity and Ecosystem Dynamics of the University of Amsterdam (UvA) in the Netherlands (NL). At the time of the IRMS analysis the age of the samples synthesized in 2017 spanned 19–24 months.

For this follow-up study, an additional set of 15 ‘fresh’ ETN samples was prepared in 2019 at TNO Technical Sciences following similar standard synthesis conditions, as described previously [3]. This set includes eight ETN samples produced *via* the mixed acid route (samples MA1, MA1a and MA2-7) and another seven ETN samples prepared *via* the nitrate salt route (samples NS1-7). A small amount (∼150–250 mg) of ETN crystals (from samples MA1, MA1 and MA2-7) were additionally used to prepare melt-cast ETN samples (samples MC1, MC1a and MC2-7) to study the effect of melt-casting on light element isotope ratios. The ETN crystals were melted around 65 °C and allowed to solidify as small casted drops. All ETN samples from 2019 were analyzed within 1–2 months after synthesis using IRMS at the UvA (NL) to determine their δ^13^C, δ^15^N, δ^2^H and δ^18^O isotope values.

### 2.3 IRMS analysis (USA)

A Thermo EA IsoLink Elemental Analyzer coupled to a Delta V Isotope Ratio Mass Spectrometer *via* a Conflo IV interface was used to determine nitrogen and carbon isotope values. The combustion reactor was packed with chromium (III) oxide, copper grains, and silvered cobaltous/cobaltic oxide (according to manufacturer’s specifications). The reactor temperature was kept at 1450 °C. After passing through a magnesium perchlorate trap for water removal, nitrogen (N_2_) and carbon dioxide (CO_2_) were separated on a packed GC column with the oven temperature set to 70 °C. Helium carrier gas and reference gas flows were set to 180 mL/min and 70 mL/min, respectively. For samples with highly oxidized nitrogen (ETN and potassium nitrate), there was no additional oxygen added for combustion [17,18]. Erythritol samples had a three second oxygen injection (flow set to 250 mL/min). Samples of erythritol (400 ± 50 μg), potassium nitrate (350 ± 50 μg) and ETN (350 ± 50 μg) were weighed into tin capsules for combustion analysis measurements. For carbon and nitrogen isotope analysis, at least five repeats were measured for each sample. l-glutamic acid standards (USGS40 and USGS41) were measured in each analytical sequence as carbon and nitrogen isotope reference materials.

### 2.4 IRMS analysis (NL)

Carbon, hydrogen, oxygen and nitrogen isotope ratios were measured using a BiovisION isotope ratio mass spectrometer (Elementar; Manchester, UK). For *δ*^13^C and *δ*^15^N isotope measurements the BiovisION was coupled to a Vario Isotope Cube (Elementar; Langenselbold, Germany) elemental analyzer (EA). The oxidation/combustion reactor tube contained silverwool and copper oxide rods. Sample residue was collected in a quartz ash finger. The reduction reactor tube was filled with aluminium oxide balls and silverwool to remove halogens and copper. Two phosphorous pentoxide chemical traps were used to remove any water generated during combustion. For *δ*^2^H and *δ*^18^O isotope measurements the IRMS was equipped with a Vario Pyro Cube EA (Elementar, Langenselbold, Germany) containing a glassy carbon pyrolysis tube filled with glassy carbon and graphite. In addition a trap was used, filled with 50% Sicapent® and 50% NaOH on a substrate, to remove acidic pyrolysis products, water and CO_2_ from the system.

Carbon and nitrogen isotope ratios were determined by detecting the CO_2_ and N_2_ gas ions. Solid samples of 3.5–5.0 mg erythritol, ±0.5 mg ETN and ±0.5 mg KNO_3_ (or other nitrate salt) were prepared in 11 × 4 × 4 mm tin boats (erythritol) and 3.3ø × 5 mm tin capsules (ETN and nitrate salts). The combustion and reduction reactor temperatures were held at 950 °C and 600 °C, respectively. Helium carrier gas flow and detector reference gas flow were set to 230 mL/min. For the ETN samples no additional oxygen was added for combustion. For the erythritol samples oxygen was dosed for 90 s at a flow rate of 70 mL/min. For carbon and nitrogen isotope analysis, three repeats were measured for each sample. The international reference materials caffeine (IAEA-600) and sucrose (IEAE-CH-60) were included for the carbon isotope analysis. Two ammonium sulfate standards (USGS-25 and IAEA-N-2) were measured in each analytical sequence as nitrogen isotope reference materials. An in-house acetanilide standard was used as quality control check for carbon and nitrogen.

Hydrogen and oxygen isotope ratios were determined from the measurement of the hydrogen (H_2_) and carbon monoxide (CO) gas ions. The high thermal conversion furnace temperature was set at 1450 °C. Solid samples were prepared in 11 × 4 × 4 mm silver boats (erythritol) and 3.3ø × 5 mm silver capsules (ETN and nitrate salts) containing ±1.5 mg erythritol, ±0.5 mg ETN and ±0.5 mg KNO_3_ (or alternative nitrate salt). For hydrogen and oxygen isotope analysis, at least four repeats were measured for each sample. Prior to analysis the erythritol samples were dried in an oven for five days at 50 °C and stored in a desiccator until use. Polyethylene (IAEA-CH-7) and two caffeine standards (USGS-62 and USGS-63) were used as hydrogen reference standards. Potassium nitrate (USGS-32) and benzoic acid (IAEA-602) were measured as oxygen isotopic reference materials. A quality control check for hydrogen and oxygen isotope measurements was performed using an in-house caffeine standard.

### 2.5 Data analysis

Isotope ratios were determined based on 2–11 repeat analyses. The included repeats varied due to outlier removal and system stabilization. The IRMS data was normalized using a two-point calibration as described by Paul et al. [19] and Sharp [20]. Offline correction was performed for all isotopic data (C, N, H and O) based on the measured isotope reference standards in each analytical sequence. The data was normalized against the Vienna Pee Dee Belemnite (VPDB) international reference scale for carbon, the Atmospheric Nitrogen (Air-N_2_) international reference scale for nitrogen, and the Vienna Standard Mean Ocean Water (VSMOW) international reference scale for hydrogen and oxygen.

The potential to differentiate between erythritol and nitrate source samples based on the IRMS data was studied by calculating the discrimination power (*DP*) as introduced by Smalldon and Moffat [21] and illustrated by Gallidabino et al. [22] using the following equation:

(1) $$DP\;(\text{\%})=100\cdot \left[1-\frac{2\cdot m}{n\cdot (n-1)}\right]$$

Where *m* is the number of undiscriminated sample pairs and *n* is the total number of samples.

For the erythritol samples the individual discrimination power was calculated for the carbon, hydrogen and oxygen isotope ratios. In addition, the overall discrimination power of the erythritol samples was calculated of the combined IRMS data. For the nitrate salt and nitric acid samples the individual discrimination power was calculated for the nitrogen and oxygen isotope data. The overall discrimination power was calculated based on the combined nitrogen and oxygen isotope ratios.

## 3 Results and discussion

### 3.1 Erythritol isotopic composition and distribution

**Fig. 1:**
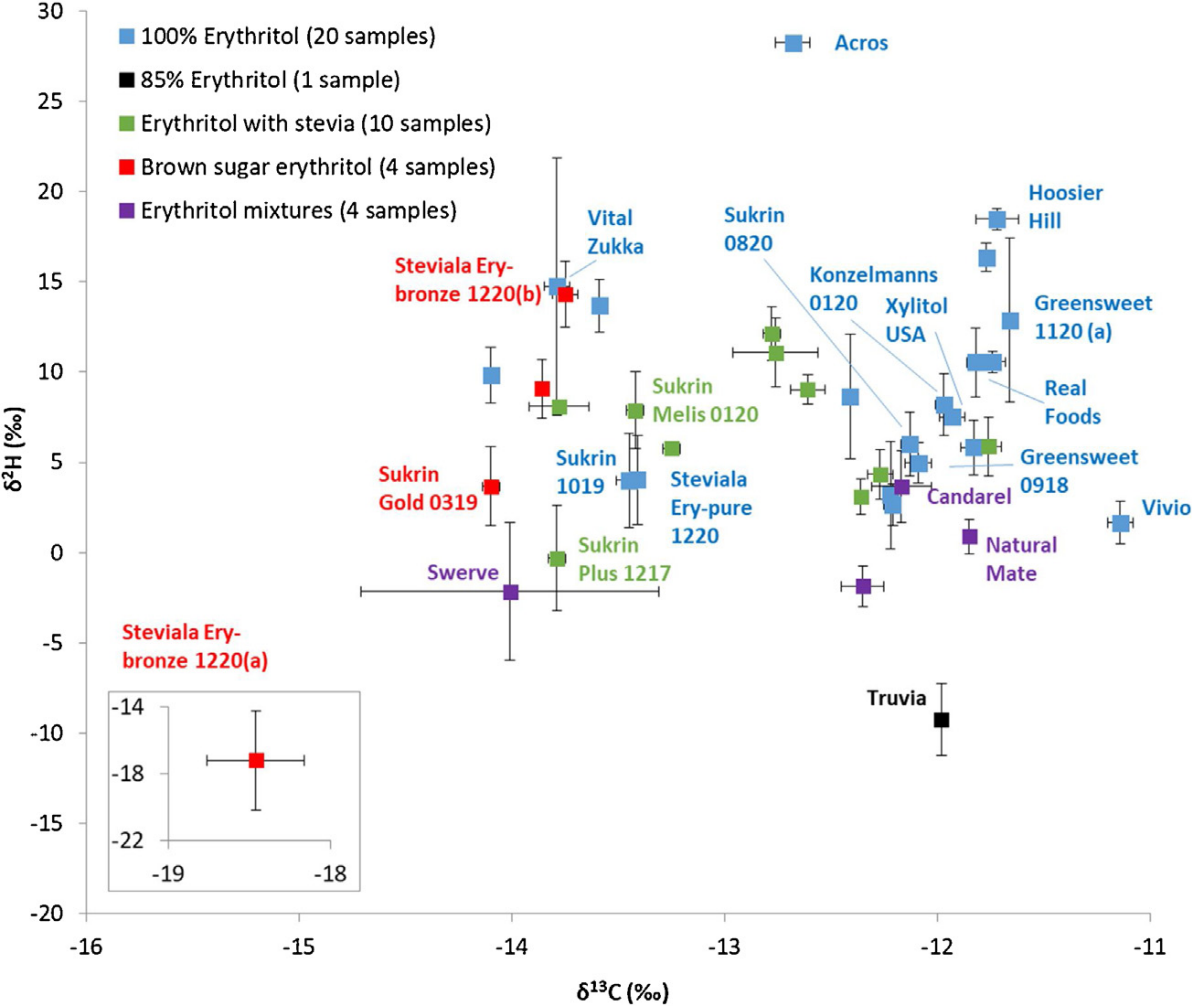
Carbon (δ^13^C) and hydrogen (δ^2^H) isotope values with ±2SD of erythritol precursors (39 samples in total). The SD values have been determined based on three repeat analyses for carbon and at least two (2-5) repeat analyses for hydrogen. The sample IDs of the erythritol precursors that were selected for ETN synthesis are given in the figure.

**Fig. 2:**
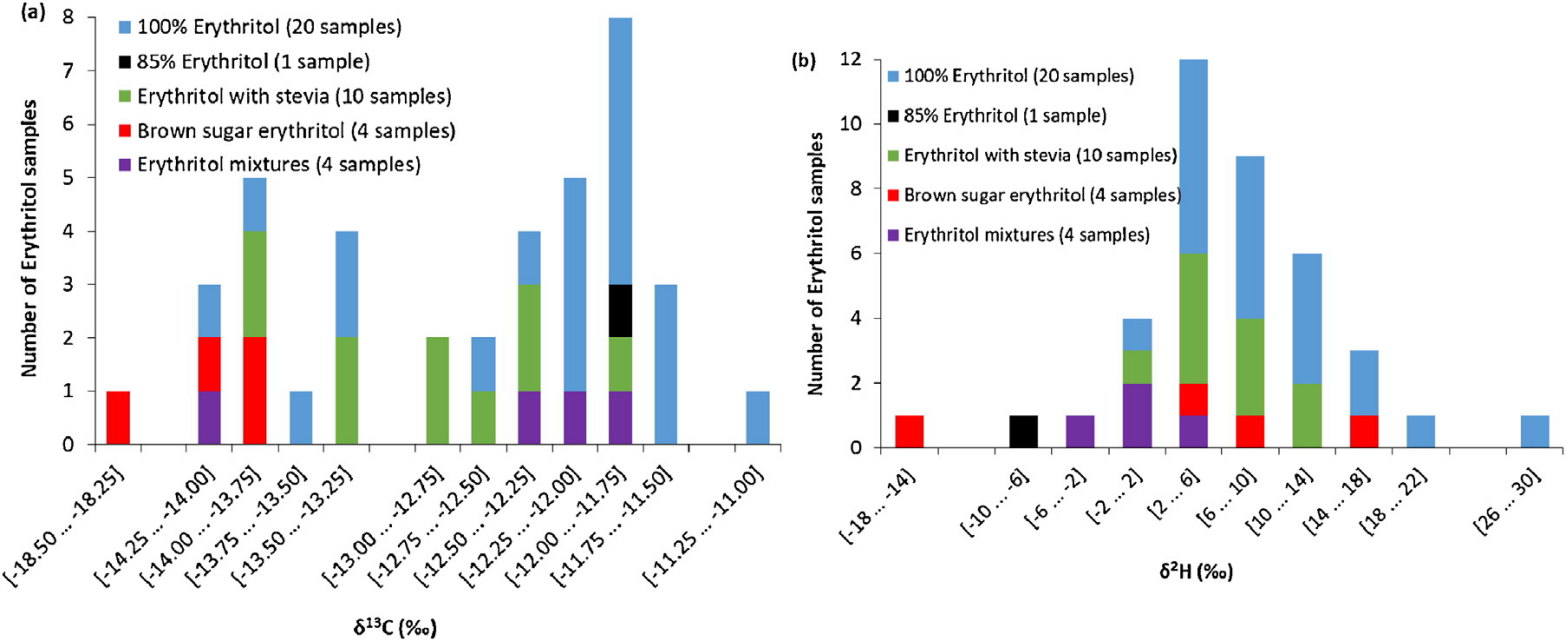
*(a)* Carbon (δ^13^C), and *(b)* hydrogen (δ^2^H) isotope distributions of the erythritol precursors (39 samples in total).

Fig. 1 shows the carbon and hydrogen isotope ratios of the erythritol samples divided in five categories. The distribution of the carbon and hydrogen results is given in Fig. 2. The δ^13^C values of the pure erythritol samples ranged from −14.10‰ to −11.14‰ (SD < 0.10‰). Because erythritol is only 70% as sweet as sugar [1], it is commonly found as mixtures with other sugar substitutes such as stevia, oligosaccharides, or other carbon-based sugar substitutes. Most erythritol mixtures (containing mixtures of erythritol with other sugar substitutes) fell within the carbon isotope range of the pure samples, with one exception for sample ‘Steviala Ery-bronze 1220(a)’. This brown sugar erythritol exhibited a more negative *δ*^13^C value of −18.46‰ (SD = 0.15‰). The ‘Swerve’ erythritol sample (erythritol mixture with oligosaccharides) fell within the upper carbon range (*δ*^13^C = −14.01‰) but had a significantly higher measurement variation (SD = 0.35‰).

For hydrogen, the *δ*^2^H values of the pure erythritol samples ranged from 1.66‰ to 28.25‰ (SD < 1.80‰, with two outliers of SD = 2.27‰ and SD = 3.56‰ for sample ‘Greensweet 1120(a)’ and sample ‘Vital Zukka’, respectively). Most erythritol mixtures fell within the range of the pure erythritol samples, but some mixtures had slightly more negative δ^2^H values down to −2.15‰. There were two erythritol mixtures, sample ‘Truvia’ (85% erythritol) and sample ‘Steviala Ery-bronze 1220(a)’, that exhibited considerably more negative δ^2^H values of −9.25‰ and −17.19‰, respectively.

The oxygen isotopic values ranged from 26.03‰ to 29.84‰ (SD < 0.50‰) for the pure erythritol. With the exception of the ‘Truvia’ sample (85% erythritol) with a δ^18^O value of 24.34‰, all other erythritol mixtures exhibit similar oxygen values as observed for the pure erythritol samples. The detailed carbon, hydrogen and oxygen isotopic compositions of the individual erythritol precursors are listed in Table S3 of the supplementary information.

The discrimination power between the erythritol samples based on their isotopic compositions has been calculated using Eq. (1). Sample pairs are defined as ‘undiscriminated’ when the isotopic value(s) under investigation are within ±2SD of each other. Based on the carbon isotope values, a total of *m* = 209 undiscriminated erythritol sample pairs were identified (out of a total of 780 sample pairs) when comparing all *n *= 39 erythritol samples. The discrimination power based on the carbon isotope values was found to be 72%. The discrimination power between erythritol samples based on hydrogen or oxygen isotope ratios is considerably lower. Discrimination powers of 42% (*m* = 429 undiscriminated sample pairs) and 21% (*m* = 586 undiscriminated sample pairs) were identified for hydrogen and oxygen, respectively. This shows that the carbon isotopic values have the highest power to differentiate two erythritol samples. Although the hydrogen and oxygen isotope ratios have a limited discrimination power, it is still very useful to include these results for sample comparison. Especially as almost no correlation was observed for the isotope data in the erythritol samples (δ^13^C *vs* δ^2^H: *r* = 0.36, δ^13^C *vs* δ^18^O: *r* = 0.32, δ^18^O *vs* δ^2^H: *r* = 0.02). According to Smalldon and Moffat [21] the combined discrimination power (*DP*) for uncorrelated features is given by:

(2) $${DP}_{k}\;(\text{\%})=100\cdot \left[1-\overset{k}{\underset{i=1}{\prod }}\left(\frac{2\cdot m_{i}}{n_{i}\cdot (n_{i}-1)}\right)\right]$$

where *k* is number of uncorrelated features that is considered in the pairwise discrimination in the dataset.

Based on the individual *DP* values, combining the δ^13^C, δ^2^H and δ^18^O data should yield an overall discrimination power of 87%. Actual pairwise comparison yielded an experimental combined *DP* value of 85% (*m *= 114 undiscriminated sample pairs) indeed showing the low degree of elemental isotopic correlation.

The carbon isotopic range observed for erythritol samples in this study of 2.96‰ (from *δ*^13^C values of −14.10‰ to −11.14‰), as shown in Fig. 2a, is considerably smaller than ranges observed for explosive precursors in other studies. An almost twice as broad δ^13^C isotope range of 5.66‰ was observed for acetone precursor samples for the formation of the explosive TATP [13], and an even broader range was observed when studying the worldwide acetone variation [23]. The erythritol precursors in this study were purchased from various countries worldwide, therefore a more elaborate study on erythritol will probably not result in a significant increase in isotope variation. The limited variation most likely originates from the fact that globally only a few companies produce erythritol on a large scale, due to the extensive investments and biotechnological expertise required. In 2016, it was estimated that the largest producer (Cargill, USA) produced 76% of all erythritol. Production plants in China accounted for the second largest output of 16% [24]. It is expected that the consumption level of erythritol will continue to grow in the next years, which could entice other companies to enter the market, leading to greater competition in the erythritol industry. This would be beneficial from a forensic perspective, potentially resulting in expanded isotopic ranges and thus a higher discrimination power.

**Fig. 3:**
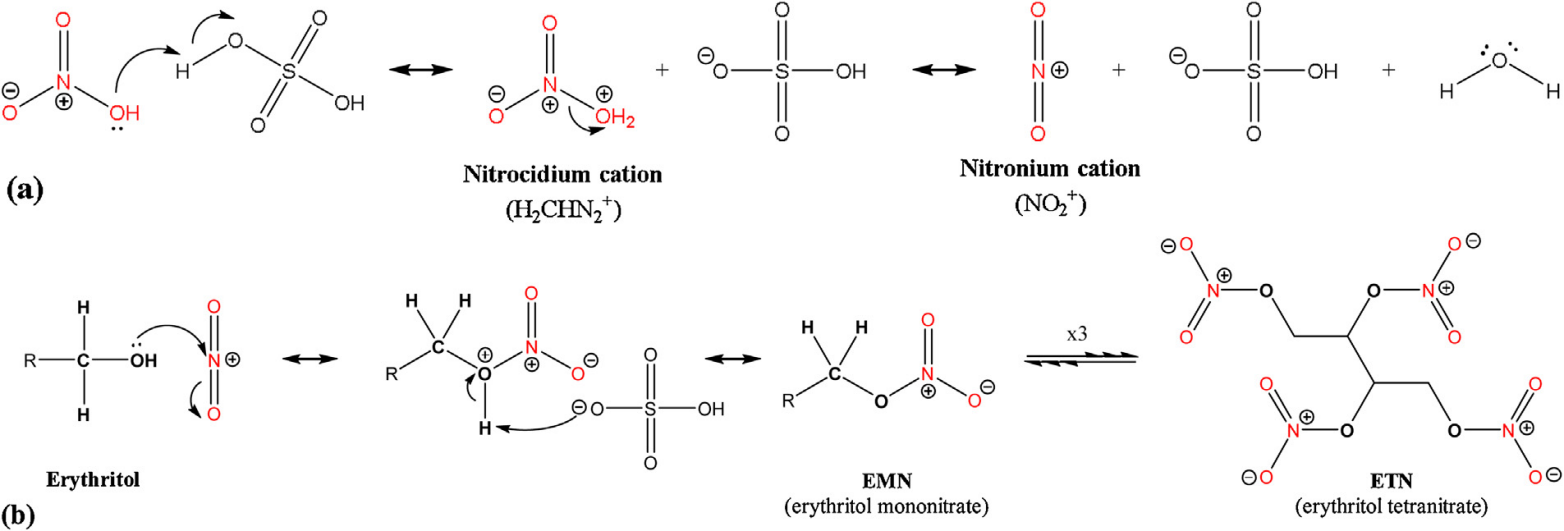
Reaction mechanism of *(a)* nitronium cation formation from nitric acid and sulfuric acid mixture, and *(b)* ETN formation from nitration of erythritol by nitric acid in an acidic aqueous environment. Red indicates atoms originating from the nitrate source (For interpretation of the references to colour in this figure legend, the reader is referred to the web version of this article).

Fig. 3 shows the postulated nitration mechanism of erythritol in the formation of ETN. The reaction mechanism shows that erythritol is the exclusive donor of carbon. Therefore, the selection of erythritol for the production of the ‘fresh’ ETN sample set is predominantly based on their carbon isotopic compositions, ensuring a broad range to study the relation between raw material and end product. The sample IDs of the erythritol precursors that were selected for the ETN-NL and ETN-USA samples in 2017 and for the ETN samples in 2019 are indicated in Fig. 1.

### 3.2 Nitrate precursor

**Fig. 4:**
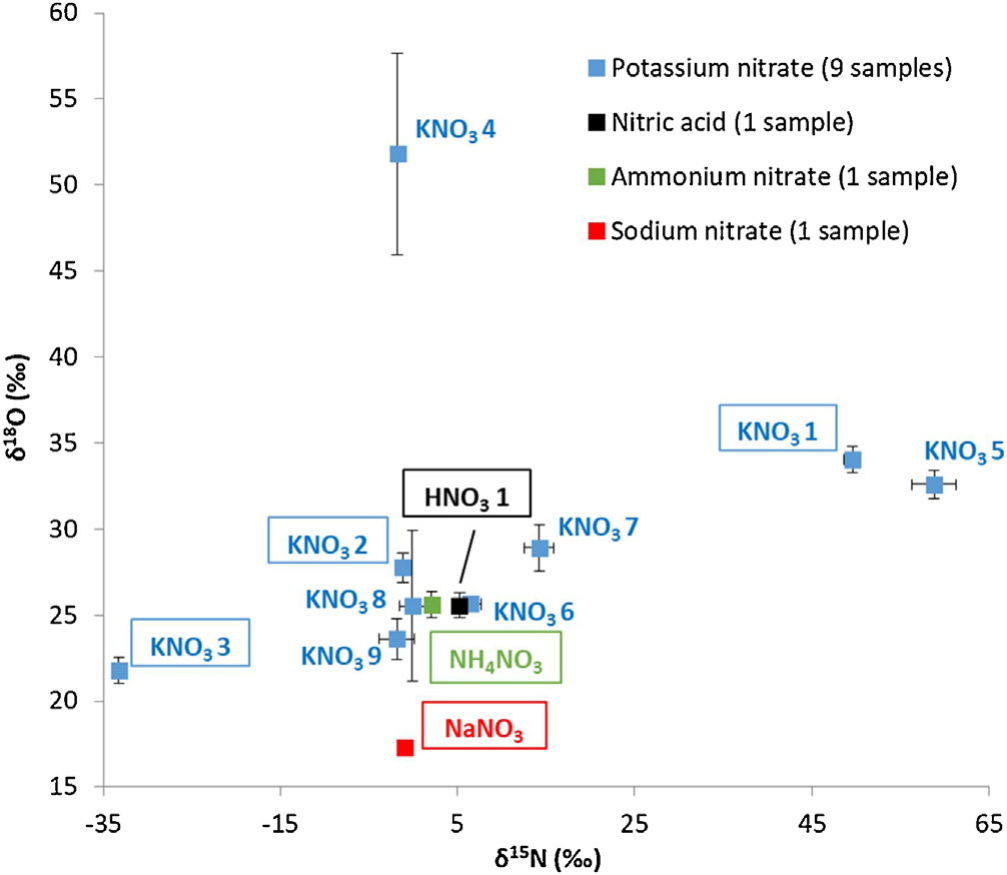
Nitrogen (δ^15^N) and oxygen (δ^18^O) isotope values with ±2SD of nitrate precursors (12 samples in total). The precursors selected for synthesis have been outlined. The SD values are based on at least three (3-9) repeat analyses for nitrogen and five repeat analyses for oxygen. No oxygen values have been determined for HNO_3_ sample 2 (δ^15^N = 4.73‰, SD = 0.13‰), HNO_3_ sample 3 (δ^15^N = 5.99‰, SD = 0.10‰), HNO_3_ sample 4 (δ^15^N = 5.96‰, SD = 0.08‰), and KNO_3_ sample 10 (δ^15^N = −1.25‰, SD = 0.10‰). Therefore, these data points are not included in the figure.

Fig. 4 presents the nitrogen and oxygen isotope values for the nitrate precursors. The precursors purchased in the USA were only measured for their δ^15^N values and are therefore these samples are not included in the graph but mentioned separately in the caption of Fig. 4. No distribution plots are provided due to the limited number of nitrate precursor samples in the dataset. The δ^15^N and δ^18^O values of the KNO_3_ samples ranged from -1.85‰ to 58.82‰ (SD < 1.00‰), and from 21.81‰ to 51.81‰ (SD < 0.70‰, with two outliers of SD = 2.19‰ and SD = 2.93‰), respectively. The measured δ^15^N values of KNO_3_ are within the previously observed ranges in other studies [9,11,14,15]. The NaNO_3_ precursor sample has a lower δ^18^O value compared to the KNO_3_ samples (17.32‰, SD = 0.22‰). The alternative nitrate salt NH_4_NO_3_ is on the lower limit of the δ^15^N isotope range observed, but in this case the δ^15^N value measured included both the nitrogen from ammonia and nitrate.

The individual discrimination powers between the nitrate samples based on the nitrogen and oxygen isotope ratios has been calculated using Eq. (1). For both isotopes a discrimination power of 74% (*m* = 17 undiscriminated pairs out of a total of 78 pairs) is obtained when comparing the nitrate samples that were measured for both nitrogen and hydrogen. Including the four nitrate precursors that were analyzed in the USA (HNO_3_ samples 2–4 and KNO_3_ sample 10) slightly lowered the discrimination power based on the nitrogen isotope values to 71%. This is a direct result of the similar nitrogen isotope ratios observed in the nitric acid samples. Similar as for erythritol almost no correlation was observed for the isotope data in the nitrate samples (δ^15^N *vs* δ^18^O: *r* = 0.30). The calculated uncorrelated overall *DP* value yielded 93%, which is slightly higher than the experimentally determined overall *DP* value of 87% based on actual pairwise comparison. However, caution has to be taken in drawing conclusions from the *DP* calculation based on only 12 nitrate samples in this study, which is too small to reflect the actual background. This might result in an overestimation of the discrimination power. Therefore, the *DP* values for the nitrate samples should be seen as indications, rather than an actual representation of the worldwide sample population.

All δ^15^N values of the HNO_3_ samples are clustered together around 5‰. Interestingly, HNO_3_ samples 3 and 4 are different bottles of nitric acid from the same lot, received at different times. Both bottles were sampled directly after opening and the isotope values are within the measurement error (5.99‰ compared to 5.96‰). To investigate the stability of the solution, HNO_3_ sample 3 was analyzed multiple times in order to track the potential change in the isotopic composition for the duration of the mixed acid synthesis. As the acid aged, and the bottle was used, the ratio of ^15^N/^14^N increased to a δ^15^N value of 6.18‰ (SD = 0.07‰) after one month, and to 6.55‰ (SD = 0.13‰) after three months. This is expected since NO_x_ fumes escape from the bottle during use and the heavier ^15^N isotopes are more likely to remain in the stock container. This illustrates that the age and use of nitric acid for the production of ETN needs to be considered in actual casework.

The nitrate precursor is the only source of nitrogen for the formation of ETN. Therefore, selection of KNO_3_ samples in this study was based on the δ^15^N isotopic values to cover the complete isotopic range. The nitrate precursors that were selected for the different ETN samples are indicated in Fig. 4 and the detailed isotopic values are listed in Table S4 of the supplementary information.

### 3.3 ETN samples

Table 1, Table 2 show the carbon and nitrogen isotope ratios of ETN-NL and ETN-USA samples synthesized using standard conditions in 2017, with δ^13^C values ranging from −18.74‰ to −11.82‰ (SD < 0.15‰, excluding two outliers), and δ^15^N values ranging from −36.31‰ to 2.94‰ (SD < 1.10‰), respectively. In addition, Table 1 includes the hydrogen and oxygen isotope ratios of the ETN-NL samples, yielding a range of −44.60‰ to −11.24‰ (SD < 2.46‰) for δ^2^H values, and 20.73‰ to 22.44‰ (SD < 0.58‰) for δ^18^O values, respectively. The isotopic compositions of the ETN samples prepared in 2019 are listed in Table 3. Carbon, hydrogen and oxygen isotope ratios showed similar ranges as observed for the 2017 samples. The nitrogen range expanded considerably, ranging from −34.07‰ to 46.52‰, caused by extension of the number of nitrate sources. The isotopic ranges observed in the ETN samples are directly related to the raw materials and is thus determined by the precursor selection in the synthesis experiments. Differences in isotopic compositions (or isotope shifts) between precursor materials and end products are caused by isotopic fractionations. These isotopic fractionations are expressed in enrichment values (ε), *i.e.,* the isotope value of the product over the isotope value of the precursor, in δ notation units (‰) [20]. Enrichment values for the ETN-NL and ETN-USA samples related to the erythritol and nitrate source precursors are listed in Table 1, Table 2, Table 3.

**Table 1:** Overview of ETN-USA 2017 sample set (12 samples in total, 4-11 repeat analyses (n) per sample) synthesized using standard synthesis conditions. The precursors used for synthesis of ETN are listed, together with the resulting yields, isotope ratios and enrichment values. *Mixed acid route standard conditions:* Add 9.3 mL of fuming nitric acid to erythritol/sulfuric acid at 0 °C, 30 min at 0 °C, 1 h at room temperature (RT) and precipitate product in ice water. *Nitrate salt route standard conditions:* Add erythritol to 10 g of nitrate salt in sulfuric acid at 15-20 °C, stir for 1 h at RT and precipitate product in ice water.

Mixed acid synthesis route (6 samples)											
	Precursors			Carbon^b^				Nitrogen^b^			
Sample ID	Erythritol	Nitrate	Yield^a^	δ^13^C	SD	n	ε^c^	δ^15^N	SD	n	ε^d^
USA-MA1	Acros	HNO_3_ 2	77.4	−12.91	0.12	10	−0.23	2.70	0.07	4	−2.02
USA-MA2	Hoosier Hill	HNO_3_ 2	71.5	−11.82	0.08	8	−0.10	2.60	0.07	5	−2.12
USA-MA3	Real Foods	HNO_3_ 2	74.1	−13.15	0.13	6	−1.35	2.62	0.14	6	−2.10
USA-MA4	Xylitol USA	HNO_3_ 2	75.6	−11.85	0.14	10	0.08	2.89	0.14	6	−3.06
USA-MA5	Swerve	HNO_3_ 2	57.6	−14.19	0.12	11	−0.18	2.55	0.13	6	−2.17
USA-MA6	Truvia	HNO_3_ 2	72.2	−12.20	0.11	10	−0.22	2.94	0.09	6	−3.01

Nitrate salt synthesis route (6 samples)											
	Precursors			Carbon^b^				Nitrogen^b^			
Sample ID	Erythritol	Nitrate	Yield^a^	δ^13^C	SD	n	ε^c^	δ^15^N	SD	n	ε^d^
USA-NS1	Acros	KNO_3_ 10	49.6	−13.29	0.09	8	−0.62	−3.70	0.10	5	−2.45
USA-NS2	Hoosier Hill	KNO_3_ 10	42.0	−12.25	0.08	5	−0.54	−4.02	0.06	6	−2.77
USA-NS3	Real Foods	KNO_3_ 10	55.4	−12.41	0.16	8	−0.60	−3.79	0.06	9	−2.54
USA-NS4	Xylitol USA	KNO_3_ 10	49.8	−12.37	0.09	8	−0.45	−3.97	0.07	7	−2.72
USA-NS5	Swerve	KNO_3_ 10	51.8	−14.89	0.10	6	−0.89	−3.95	0.14	9	−2.70
USA-NS6	Truvia	KNO_3_ 10	24.4	−12.33	0.14	8	−0.35	−4.40	0.14	9	−3.15

a Yields are expressed in %.

b Isotopic values and enrichments (ε) are expressed in ‰.

c ETN enrichment *versus* erythritol precursor.

d ETN enrichment *versus* nitrate precursor.

**Table 2:** Overview of ETN-NL 2017 sample set (24 samples in total, 2-5 repeat analyses (n) per sample) synthesized using standard synthesis conditions. The precursors used for synthesis of ETN are listed, together with the resulting yields, isotope ratios and enrichment values. *Mixed acid route standard conditions:* Add 9.3 mL of fuming nitric acid to erythritol/sulfuric acid at room temperature (RT), 1 h at 35 °C and precipitate product in ice water. *Nitrate salt route standard conditions*: are provided in Table 1.

Mixed acid synthesis route (11 samples)																		
	Precursors			Carbon^b^(n = 3)			Nitrogen^b^(n = 3)			Hydrogen^b^				Oxygen^b^				
Sample ID	Erythritol	Nitrate	Yield^a^	δ^13^C	SD	ε^c^	δ^15^N	SD	ε^d^	δ^2^H	SD	n	ε^c^	δ^18^O	SD	n	ε^c^	ε^d^
NL-MA1	Candarel	HNO_3_ 1	49.0	−12.36	0.04	−0.19	1.37	0.04	−3.84	−22.51	1.13	4	−26.08	21.38	0.14	5	−6.61	−4.09
NL-MA2	Steviala Ery-pure 1220	HNO_3_ 1	49.4	−13.43	0.29	0.02	0.74	0.05	−4.47	−11.90	0.84	4	−15.84	21.74	0.37	5	−6.94	−3.73
NL-MA3A	Sukrin 1019	HNO_3_ 1	52.6	−13.65	0.05	−0.24	1.84	0.28	−3.37	−11.98	1.04	5	−15.95	21.72	0.12	5	−7.16	−3.75
NL-MA3B	Sukrin 1019	HNO_3_ 1	48.6	−13.69	0.05	−0.28	1.04	0.15	−4.17	−17.55	1.24	3	−21.49	–	–	–	–	–
NL-MA4	Greensweet 0918	HNO_3_ 1	58.6	−12.43	0.11	−0.34	1.43	0.19	−3.78	−23.41	1.70	3	−28.23	22.44	0.09	3	−7.19	−3.05
NL-MA5	Konzelmanns 0120	HNO_3_ 1	58.4	−12.27	0.10	−0.30	1.59	0.09	−3.62	−26.74	2.09	5	−34.67	21.81	0.19	5	−7.18	−3.67
NL-MA6	Steviala Ery-bronze 1220(a)	HNO_3_ 1	51.4	−18.59	0.02	−0.13	1.28	0.07	−3.93	−43.39	1.53	2	−26.66	21.55	0.58	4	−6.78	−3.92
NL-MA7	Sukrin Gold 0319	HNO_3_ 1	47.0	−13.99	0.03	0.11	1.10	0.09	−4.11	−13.68	2.23	4	−17.29	21.31	0.42	5	−7.56	−4.15
NL-MA8	Sukrin Plus 1217	HNO_3_ 1	43.0	−13.96	0.01	−0.17	0.90	0.20	−4.31	−24.34	0.78	2	−24.04	21.53	0.15	5	−6.83	−3.94
NL-MA9	Natural Mate	HNO_3_ 1	51.8	−12.19	0.05	−0.34	1.32	0.01	−3.89	−36.15	2.46	2	−37.01	20.73	0.09	5	−5.85	−4.72
NL-MA10	Sukrin Melis 0120	HNO_3_ 1	58.8	−13.76	0.07	−0.35	1.15	0.04	−4.06	−11.24	0.70	4	−18.98	21.78	0.13	5	−7.29	−3.70

Nitrate salt synthesis route (13 samples)																		
	Precursors			Carbon^b^(n = 3)			Nitrogen^b^(n = 3)			Hydrogen^b^				Oxygen^b^				
Sample ID	Erythritol	Nitrate	Yield^a^	δ^13^C	SD	ε^c^	δ^15^N	SD	ε^d^	δ^2^H	SD	n	ε^c^	δ^18^O	SD	n	ε^c^	ε^d^
NL-NS1	Candarel	KNO_3_ 3	36.0	−12.69	0.01	−0.53	−35.99	0.09	−2.83	−29.02	2.37	3	−32.57	21.81	0.36	5	−6.20	0.00
NL-NS2	Steviala Ery-pure 1220	KNO_3_ 3	37.8	−13.97	0.05	−0.53	–	–	–	−16.87	2.03	4	−20.79	21.84	0.09	5	−6.84	0.03
NL-NS3A	Sukrin 1019	KNO_3_ 3	43.2	−13.76	0.02	−0.35	−36.00	0.08	−2.84	−15.42	0.23	4	−19.37	22.36	0.01	4	−6.54	0.54
NL-NS3B	Sukrin 1019	KNO_3_ 3	40.0	−14.19	0.27	−0.79	−36.31	0.08	−3.17	−13.65	1.70	4	−17.61	21.64	0.12	5	−7.24	−0.17
NL-NS4	Greensweet 0918	KNO_3_ 3	40.6	−12.60	0.06	−0.52	−35.77	0.10	−2.61	−26.66	1.64	3	−31.46	21.93	0.24	5	−7.68	0.12
NL-NS5	Konzelmanns 0120	KNO_3_ 3	49.0	−12.38	0.15	−0.41	−36.22	1.10	−3.07	−29.13	0.45	5	−37.04	21.68	0.14	5	−7.31	−0.13
NL-NS6	Steviala Ery-bronze 1220(a)	KNO_3_ 3	45.4	−18.74	0.06	−0.29	−35.81	−0.05	−2.65	−44.60	2.26	2	−27.89	21.24	0.52	5	−7.08	−0.56
NL-NS7	Sukrin Gold 0319	KNO_3_ 3	40.2	−14.21	0.08	−0.11	−35.68	0.07	−2.51	−22.40	1.88	2	−25.97	21.85	0.07	5	−7.04	0.04
NL-NS8	Sukrin Plus 1217	KNO_3_ 3	28.6	−14.93	0.10	−1.16	−35.74	0.11	−2.58	−22.31	0.41	4	−22.01	22.24	1.12	4	−6.14	0.42
NL-NS9	Natural Mate	KNO_3_ 3	15.0	−12.57	0.04	−0.73	−35.90	0.01	−2.74	−34.50	0.39	2	−35.36	21.16	0.05	5	−5.43	−0.64
NL-NS10	Sukrin Melis 0120	KNO_3_ 3	25.8	−13.95	0.14	−0.54	−35.94	0.08	−2.78	–	–	–	–	22.06	0.15	5	−7.01	0.24
NL-NS11	Sukrin 1019	NaNO_3_	14.2	−14.38	0.38	−0.98	−2.98	0.25	−2.03	−10.06	2.61	3	−14.03	21.83	0.20	5	−7.05	4.43
NL-NS12	Sukrin 1019	NH_4_NO_3_	23.2	−14.39	0.20	−0.99	−0.21	0.16	−2.30	−14.12	0.45	4	−18.08	23.31	0.27	4	−5.62	−2.22

a Yields are expressed in %.

b Isotopic values and enrichments (ε) are expressed in ‰.

c ETN enrichment *versus* erythritol precursor.

d ETN enrichment *versus* nitrate precursor.

**Table 3:** Overview of ETN 2019 sample set (15 samples in total, 2-5 repeat analyses (n) per sample) synthesized using standard synthesis conditions. The precursors used for synthesis of ETN are listed, together with the resulting yields, isotope ratios and enrichment values. *Mixed acid and nitrate salt route standard synthesis conditions*: are provided in Table 2.

Mixed acid synthesis route (8 samples)																	
	Precursors			Carbon^b^(n = 3)			Nitrogen^b^(n = 3)			Hydrogen^b^				Oxygen^b^(n = 4)			
Sample ID	Erythritol	Nitrate	Yield^a^	δ^13^C	SD	ε^c^	δ^15^N	SD	ε^d^	δ^2^H	SD	n	ε^c^	δ^18^O	SD	ε^c^	ε^d^
MA1	Sukrin 1019	HNO_3_ 1	47.6	−13.66	0.07	−0.25	2.57	0.04	−2.65	−8.16	1.62	2	−12.14	22.55	0.16	−6.36	−2.94
MA1a	Sukrin 1019	HNO_3_ 1	46.8	−13.74	0.06	−0.33	2.32	0.01	−2.89	−9.31	0.40	4	−13.29	22.54	0.15	−6.36	−2.95
MA2	Sukrin 0820	HNO_3_ 1	61.4	−12.54	0.01	−0.42	2.29	0.01	−2.92	−22.08	0.79	2	−27.91	22.38	0.44	−6.11	−3.11
MA3	Steviala Ery-bronze 1220(a)	HNO_3_ 1	49.6	−18.66	0.05	−0.20	2.24	0.02	−2.97	−42.37	0.56	2	−25.62	22.12	0.42	−6.22	−3.36
MA4	Steviala Ery-bronze 1220(b)	HNO_3_ 1	59.6	−13.93	0.04	−0.18	2.29	0.01	−2.92	−15.33	3.27	2	−29.21	22.40	0.09	−6.39	−3.09
MA5	Vivio	HNO_3_ 1	57.4	−11.45	0.04	−0.31	2.36	0.02	−2.86	−32.00	2.43	2	−33.60	20.82	0.28	−5.51	−4.63
MA6	Acros	HNO_3_ 1	31.2	−12.50	0.01	0.18	2.67	0.05	−2.55	−9.93	0.13	2	−37.13	21.99	0.12	−3.94	−3.49
MA7	Real Foods	HNO_3_ 1	50.8	−11.90	0.07	−0.08	2.49	0.09	−2.73	−15.29	0.59	2	−25.55	21.59	0.28	−5.80	−3.88

Nitrate salt synthesis route (7 samples)																	
	Precursors			Carbon^b^(n = 3)			Nitrogen^b^(n = 3)			Hydrogen^b^				Oxygen^b^(n = 4)			
Sample ID	Erythritol	Nitrate	Yield^a^	δ^13^C	SD	ε^c^	δ^15^N	SD	ε^d^	δ^2^H	SD	n	ε^c^	δ^18^O	SD	ε^c^	ε^d^
NS1	Sukrin 1019	KNO_3_ 3	31.8	−13.76	0.04	−0.35	−34.07	0.06	−0.85	−14.59	1.50	4	−18.55	22.85	0.15	−6.06	1.02
NS2	Sukrin 1019	KNO_3_ 1	39.6	−13.89	0.03	−0.49	46.51	0.19	−2.94	−12.08	1.37	4	−16.05	23.30	0.37	−5.63	−10.38
NS3	Sukrin 1019	KNO_3_ 2	38.6	−13.78	0.03	−0.38	−2.87	0.06	−1.70	−11.58	2.29	5	−15.55	22.97	0.09	−5.95	−4.65
NS4	Acros	KNO_3_ 3	40.2	−13.04	0.01	−0.36	−33.84	0.06	−0.61	−8.25	2.19	4	−35.50	22.14	0.11	−3.79	0.32
NS5	Real Foods	KNO_3_ 1	40.4	−12.19	0.04	−0.37	46.46	0.10	−2.99	−14.37	0.84	3	−24.64	23.46	0.19	−3.98	−10.22
NS6	Vivio	KNO_3_ 1	45	−11.61	0.05	−0.48	46.52	0.07	−2.93	−34.28	1.54	2	−35.88	22.64	0.24	−3.74	−11.02
NS7	Sukrin 0820	KNO_3_ 2	41	−12.55	0.08	−0.43	−2.86	0.04	−1.69	−28.42	1.82	3	−34.21	22.88	0.59	−5.63	−4.74

a Yields are expressed in %.

b Isotopic values and enrichments (ε) are expressed in ‰.

c ETN enrichment *versus* erythritol precursor.

d ETN enrichment *versus* nitrate precursor.

The ETN samples NL-MA3A and NL-MA3B, samples NL-NS3A and NL-NS3B, and samples MA1 and MA1a, can be considered as synthesis replicates since they were synthesized using similar conditions and precursors in the same time frame. For most of these replicates the variation in the observed isotope ratios is within the measurement uncertainty, with the exception of slightly higher variations for the carbon composition of ETN samples NL-NS3A and NL-NS3B (sample variation of 0.30‰ *versus* an observed measurement variation of SD < 0.15‰), and the hydrogen composition of ETN samples NL-MA3A and NL-MA3B (sample variation of 3.94‰ *versus* a measurement variation of SD < 2.46‰).

ETN samples NL-MA3A and NL-MA3B, and samples MA1 and MA1a were prepared with similar erythritol and nitrate precursors with an interval of two years. The carbon isotopic compositions of all four samples are within the confidence interval (2xSD), indicating that the isotopic composition of the raw materials was unaltered and that the isotopic shifts occurring during the nitration reaction are reproducible. Similar δ^13^C isotope ratios were found for all other ETN samples that were synthesized in 2017 and 2019 with the same erythritol precursor, *e.g.,* ETN sample NL-NS3A v*ersus* ETN sample NS1 (erythritol sample ‘Sukrin 1019’) and ETN sample NL-MA6 v*ersus* ETN sample MA3 (erythritol sample ‘Steviala Ery-bronze 1220(a)). Interestingly, the nitrogen isotopic composition slightly increased between the samples from 2017 and the samples from 2019. The nitric acid bottle used in 2019 was already opened during the ETN synthesis in 2017, so the observed change in δ^15^N value is in line with the change observed for aged acid in section 3.2. The results showed that using a bottle of nitric acid that has been open for two years results in nitrogen isotopic values that are 1.0–1.5‰ higher than the initial nitrogen isotopic value. Additionally, the nitrate isotope value of ETN sample NS1 (2019) is almost 2‰ more positive than that of ETN sample NL-NS-3A (2017), synthesized *via* the nitrate salt route with a similar KNO_3_ source.

Because of its relatively low melting point ETN can be melt-casted to produce miniaturized constructions (or shapes and forms) that blend well in the environment and do not stand out as an IED. As this is occasionally observed at the NFI in casework, the effect of melt-casting on the isotope ratios was studied. Melt-cast ETN samples were prepared from small amounts of ETN crystals originating from samples synthesized *via* the mixed acid route in 2019 (ETN samples MA1-7). The carbon, nitrogen, hydrogen and oxygen values of these melt-cast ETN samples are listed in Table S5 of the supplementary information. All isotopic compositions remain constant compared to their original ETN sample (*e.g.,* sample MA1 *versus* sample MC1). This demonstrates that IRMS data of melt-casted samples in casework can directly be compared to potential ETN sources and associated raw materials.

In addition to standard synthesis conditions, ETN samples were prepared in 2017 using different synthesis conditions. Replacing laboratory-grade sulfuric acid with battery acid (ETN samples NL-MA13 and NL-NS14) had no effect on the carbon, nitrogen and hydrogen isotope values of the ETN product. For oxygen significant higher δ^18^O values of 26.25‰ and 29.33‰ (for ETN samples NL-MA13 and NL-NS14, respectively) were found using battery acid, compared to the δ^18^O values of −21.72‰ and 22.36‰ (for NL-MA3A and NL-NS3A, respectively) for ETN prepared with laboratory-grade sulfuric acid. This indicates that the oxygen isotopic composition of the sulfuric acid influences the oxygen isotope value of the ETN product, which might be caused by exchange of oxygen in the formation of the nitronium cation (Fig. 3a). Varying post-synthesis work-up conditions, including different washing and recrystallization solutions, did not have any influence on the isotopic composition of the ETN samples. The isotopic results of the ETN-NL samples synthesized under varying conditions can be found in Table S6 of the supplementary information.

ETN samples were also prepared with various reaction times. The effect on the carbon and nitrogen isotopic values is shown in Table 4. There is little variation in the *δ*^15^N values of the ETN samples for both the mixed acid and the nitrate salt route. In the mixed acid synthesis route, the *δ*^13^C values are more positive with longer reaction times (−11.73‰ at 6 h compared to −12.20‰ at 1 h). Allowing the synthesis mixture to react longer pushed the carbon isotopic composition of the ETN product closer to the *δ*^13^C value of the erythritol precursor (−11.98‰), resulting in a reduced enrichment factor. In the example of Table 4, the erythritol precursor ‘Truvia’ is a mixture containing 85% erythritol. Therefore, the final positive enrichments observed at longer reaction times are a combination of less negative enrichment for the erythritol in the precursor mixture and the carbon isotope composition of the additional 15% components in the mixture.

**Table 4:** Overview of δ^13^C and δ^15^N isotope values of ETN (9 samples in total, 5-10 repeat analyses (n) per sample) prepared with various synthesis times. For these non-standard syntheses, the ‘Truvia’ erythritol mixture was used as the erythritol source. MA and NS indicate synthesis according to the mixed acid and nitrate salt route, respectively.

Varying synthesis times (9 samples)										
			Carbon^b^				Nitrogen^b^			
Sample ID	Reaction time	Yield^a^	δ^13^C	SD	n	ε^c^	δ^15^N	SD	n	ε^d^
USA-MA6	Standard (1 h)	72.2	−12.20	0.11	10	−0.22	2.94	0.09	6	−3.01
USA-MA8	2h	82.9	−11.66	0.06	5	0.32	3.39	0.05	6	−2.56
USA-MA9	4h	78.4	−11.57	0.10	5	0.41	3.36	0.04	6	−2.59
USA-MA10	6h	79.6	−11.73	0.10	6	0.25	3.52	0.07	6	−2.44
USA-NS6	Standard (1 h)	24.4	−12.33	0.14	8	−0.35	−4.40	0.14	9	−3.15
USA-NS7	0.5h	47.1	−12.08	0.10	5	−0.10	−4.03	0.06	5	−2.78
USA-NS8	2h	17.4	−12.16	0.06	6	−0.18	−3.89	0.05	6	−2.64
USA-NS9	4h	18.0	−12.08	0.07	6	−0.10	−4.14	0.09	6	−2.89
USA-NS10	6h	52.0	−12.11	0.11	5	−0.13	−3.93	0.05	6	−2.68

a Yields are expressed in %.

b Isotopic values and enrichments (ε) are expressed in ‰.

c ETN enrichment *versus* erythritol precursor.

d ETN enrichment *versus* nitrate precursor.

### 3.4 Precursor correlation and isotopic fractionation

**Fig. 5:**
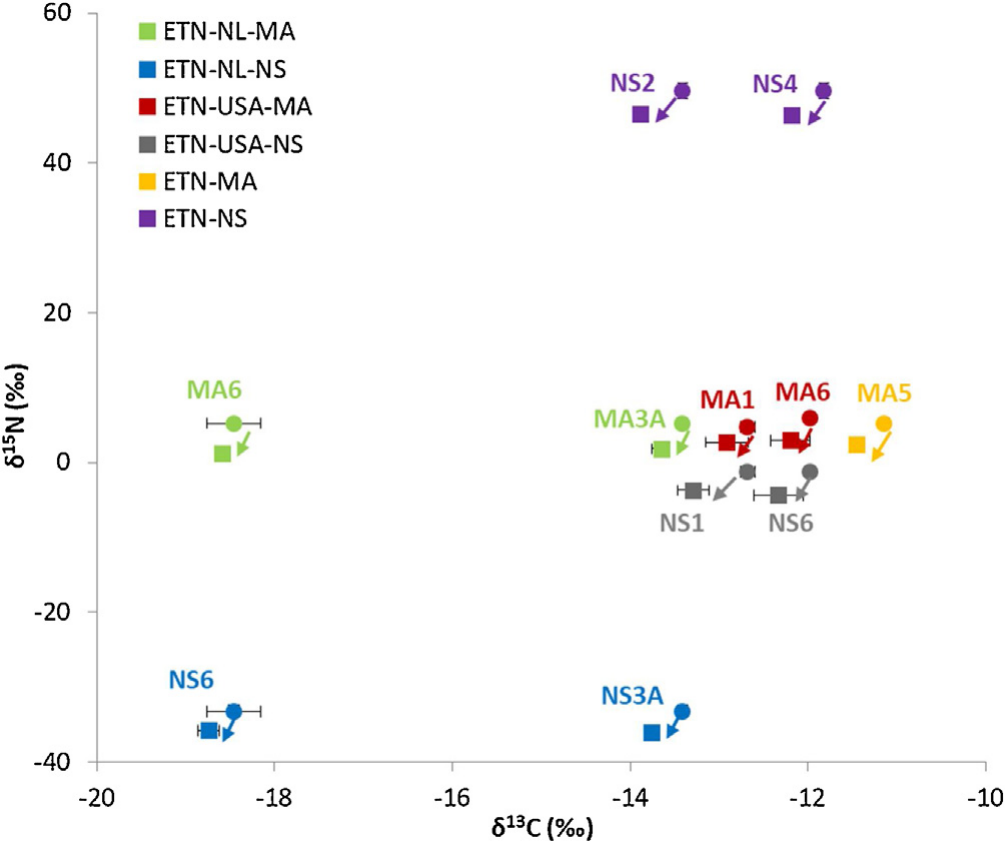
Carbon (δ^13^C) and nitrogen (δ^15^N) isotope ratios with ±2SD of ETN samples (■) and their corresponding precursors (●). The δ^13^C value corresponds to the erythritol starting material and the δ^15^N value relates to the nitrate source starting material. With MA indicating the mixed acid synthesis route, NS indicating the nitrate salt synthesis route, NL indicating production by the Dutch team, and USA indicating production by the North American team.

**Fig. 6:**
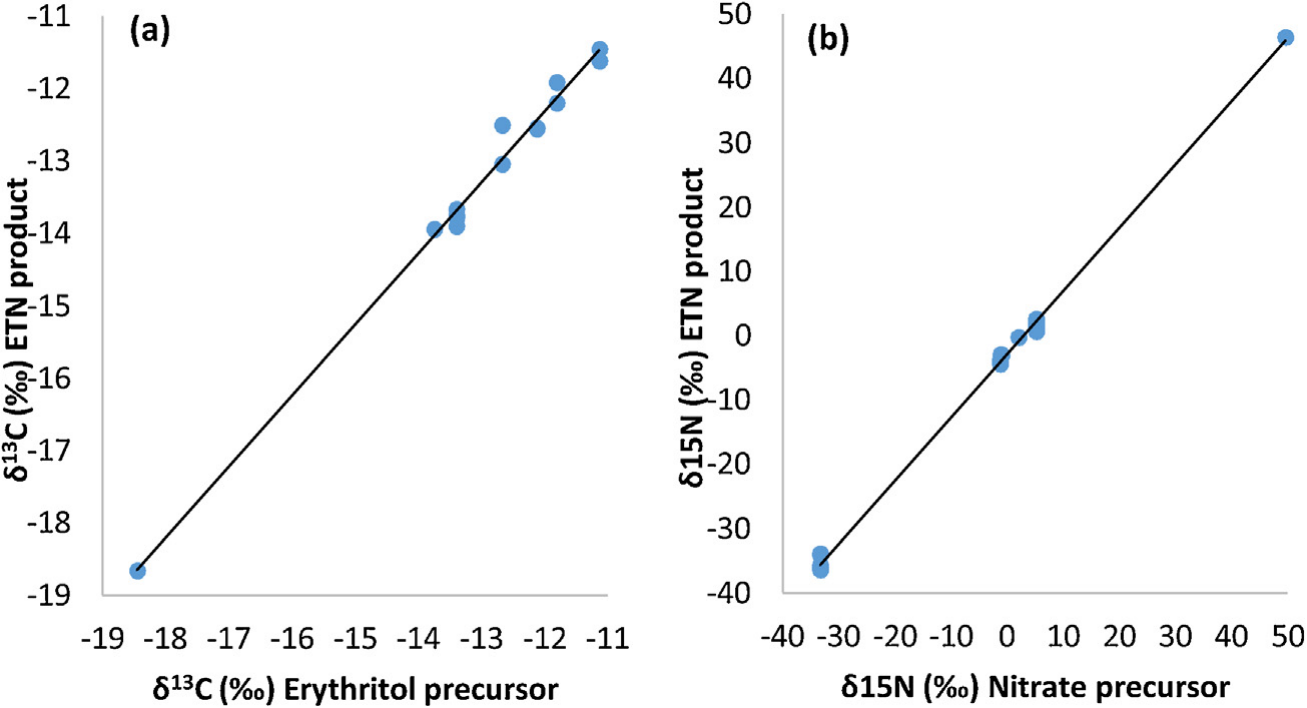
Isotope values of precursors *versus* ETN product to study the correlation of *(a)* δ^13^C values of erythritol *versus* ETN 2019 (15 samples), and *(b)* δ^15^N values of nitrate sources (KNO_3_, HNO_3_ and alternative nitrate salts) *versus* ETN-USA 2017, ETN-NL 2017 and ETN 2019 (44 samples). Correlation formula (a): δ^13^C (ETN) = 0.9825 × δ^13^C (erythritol precursor) − 0.5206, with R^2^ = 0.99 Correlation formula (b): δ^15^N (ETN) = 0.9849 × δ^15^N (nitrate precursor) − 2.9641, with R^2^ = 0.99.

Fig. 5 shows the carbon and nitrogen isotope ratios of some selected ETN samples together with the associated δ^13^C value of erythritol and δ^15^N value of the nitrate precursor combined. There is a slight shift in carbon isotopic ratio observed in the ETN product compared to the erythritol starting material. The δ^13^C value of the ETN samples becomes slightly more negative compared to its erythritol precursor, hence there is a negative enrichment (ε). This negative fractionation indicates that the lighter ^12^C isotope is easier incorporated into the ETN product and the heavier ^13^C isotope preferably remains in the unreacted fraction of the erythritol precursor. A linear relationship (R^2^ = 0.99) is observed for the δ^13^C value of ETN synthesized in 2019 *versus* erythritol, as shown in Fig. 6a. When the samples synthesized in 2017 are included, which contain more erythritol variations and mixtures, this resulted in a linear relationship of R^2^ = 0.97 (Fig. S1 of the supplementary information). This strong correlation is not surprising since erythritol is the only carbon source in the synthesis reaction (Fig. 3). The average negative enrichment is approximately −0.37‰ (SD = 0.28‰). There are a few ETN samples with a positive enrichment up to 0.18‰, but these fall within the confidence interval of the measurements. If the reaction would reach completion (100% yield) the carbon isotope ratio of ETN should be equal to that of the erythritol, because each carbon atom present in the starting material is now incorporated in the ETN explosive. Of course, this only applies for erythritol samples that are very pure. However, full conversion is never achieved in real-case scenarios, therefore a (small) negative shift will mostly be encountered. Positive shifts exceeding the measurement uncertainty are not expected, unless the positive change is due to loss of the non-erythritol components in erythritol mixtures.

When comparing the δ^15^N values of the ETN samples made by either the mixed acid or the nitrate salt route, the ETN product has a lower δ^15^N value than its nitrate precursor. Similar as for carbon, this indicates that the ETN product contains less ^15^N isotopes than the precursor material. Fig. 6b shows the robust linear relationship (R^2^ = 0.99) for the nitrogen isotopic ratios of ETN *versus* nitrate precursor for all samples prepared in 2017 and 2019. The reaction mechanism in Fig. 3 shows that the nitrate precursor is the only donor of nitrogen in the production of ETN. A maximum negative enrichment of −4.45‰ was observed for ETN samples synthesized in 2017 with ‘fresh’ nitric acid and a minimum negative enrichment of −2.55‰ was observed for ETN samples prepared with ‘2-years old’ nitric acid. The nitrogen isotopic fractionation of the ETN samples synthesized *via* the nitrate salt route ranged from −0.61‰ to −4.40‰, depending on the source of nitrate salt.

For hydrogen, a strong negative enrichment is observed between the δ^2^H value of erythritol precursor and the δ^2^H value of the ETN product. The isotopic shifts ranged between −37.13‰ and −12.14‰ (SD < 2.46‰) for all ETN samples (Table 2, Table 3). The postulated reaction mechanism in Fig. 3 suggests that the hydrogen isotope ratio in ETN samples is partly determined by the hydrogen composition of the erythritol precursor. Although all the hydrogen in the ETN molecule originates from the starting material, the hydrogen atoms in the OH groups of erythritol are replaced by NO_2_ groups upon nitration. In addition, the acidic environment might also lead to exchange phenomena and as a result no clear relation between the δ^2^H value of ETN and the corresponding erythritol precursor was found, as illustrated in Fig. S2 of the supplementary information. In the mixed acid route, the sulfuric and nitric acid can react prior to nitration of erythritol. Since sulfuric acid is the stronger acid it protonates nitric acid, forming nitracidium cations (H_2_ONO_2_^+^), which reacts further into nitronium cations (NO_2_^+^) by removal of water (Fig. 3) [25]. In the nitrate salt route, nitric acid and hence its nitronium cation are formed by an equilibrium reaction between the nitrate salt XNO_3_ and sulfuric acid, where X is the corresponding cation, which should not precipitate in the presence of sulfate ions (*e.g.,* KNO_3_, NaNO_3_, NH_4_NO_3_) [26]. This results in milder nitrating conditions relative to the mixed acid route. During the nitration of the alcohol groups (O-nitration) of erythritol water is formed, which dilutes the acidic mixture. Therefore, another function of sulfuric acid is to serve as a dehydrating agent to avoid dilution and prevent an equilibrium shift towards the starting materials [25]. In this study the hydrogen isotope values of the nitric acid precursors and the different sulfuric acids used are unknown and therefore the effect on the δ^2^H values of the ETN samples cannot be accurately explained.

**Fig. 7:**
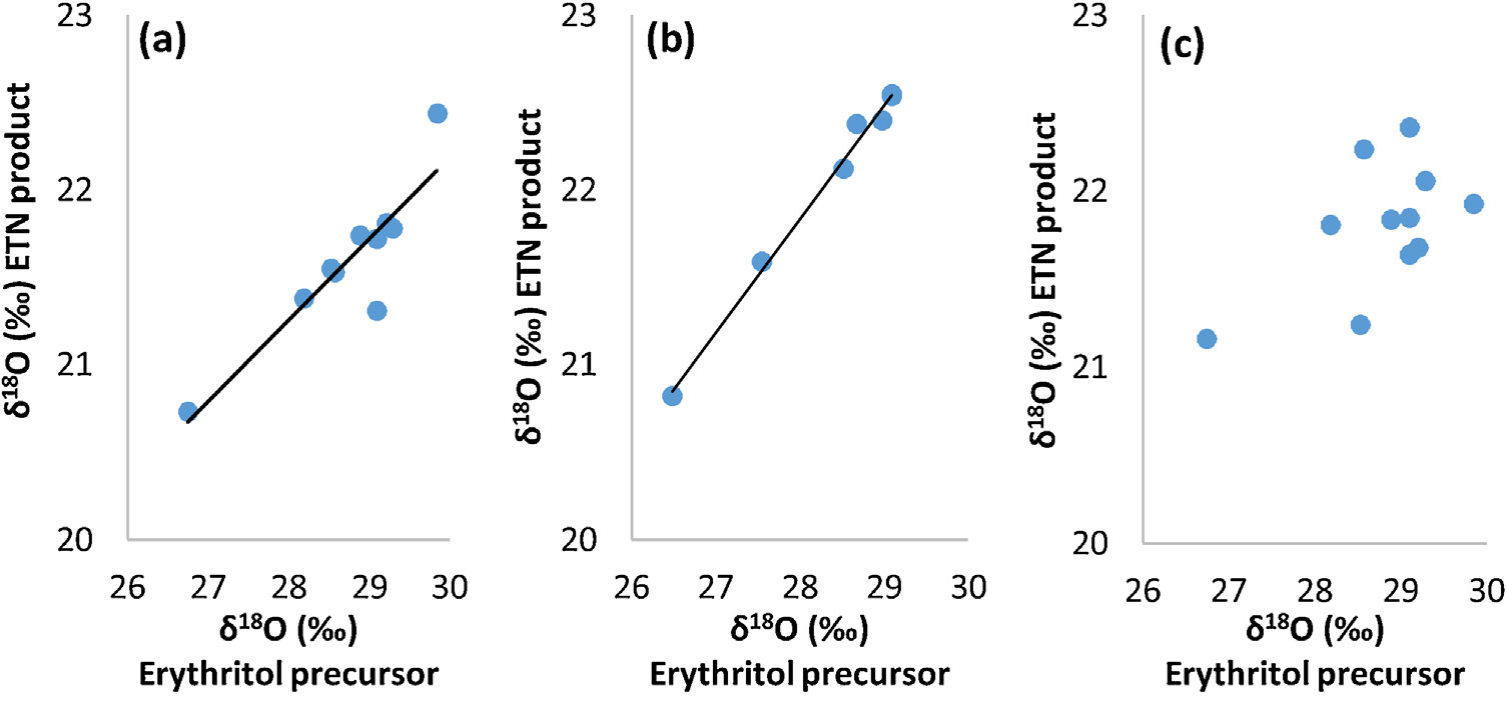
Oxygen isotope values of precursors *versus* ETN product to study the correlation of erythritol *versus (a)* ETN-NL 2017 (10 samples) synthesized with the mixed acid route (R^2^ = 0.81), *(b)* ETN 2019 (7 samples) synthesized with the mixed acid route (R^2^ = 0.99), and *(c)* ETN 2017 (11 samples) synthesized with the nitrate salt route.

The reaction mechanism of ETN formation (Fig. 3) shows that the oxygen isotope value of the ETN product is a combination of the δ^18^O values of the erythritol and the nitrate precursor. Significant negative oxygen isotopic fractionation is observed compared to the δ^18^O value of the erythritol precursor. Enrichment values are listed in Table 2, Table 3 and range from −7.68‰ to −3.74‰ (SD < 0.58‰). In the mixed acid route all samples are prepared with a similar nitric acid source (sample HNO_3_ 1), which makes it possible to study the influence of the erythritol precursor. Fig. 7a/b indicates linear relationships (R^2^ = 0.81 and R^2^ = 0.99, respectively, when excluding outlier MA6) for ETN samples prepared in 2017 and 2019. The enrichment values related to the HNO_3_ 1 precursor ranged from −4.72‰ to −2.94‰ (SD < 0.58‰). Unfortunately, the very small range of oxygen isotope values in the erythritol precursor combined with the δ^18^O value of this single nitric acid precursor hamper an accurate reconstruction of the oxygen isotopic fractionation effect in ETN synthesis.

Interestingly, ETN samples prepared in 2017 *via* the nitrate salt route using a similar potassium nitrate source (sample KNO_3_ 3) showed a poor fit to linearity (R^2^ = 0.33) between the δ^18^O values of ETN and erythritol, as seen in Fig. 7c. This might suggest a less reproducible fractionation effect during the synthesis of ETN under the milder nitrating conditions of the nitrate salt route *versus* the more aggressive mixed acid route. In addition, aging of the 2017 ETN samples might have influenced the linearity in Fig. 7a/c. Oxygen enrichment values of the ETN NL-NS samples, listed in Table 2, ranged from −7.68‰ to −5.43‰ (SD < 0.52‰) related to the erythritol starting material. The oxygen enrichment values related to KNO_3_ sample 3 (δ^18^O value of 21.81‰, SD = 0.4‰) averaged around −0.01‰ (SD = 0.36‰). In 2019, ETN NS samples were prepared using combinations of different erythritol and KNO_3_ precursors. The use of different nitrate salt precursors affected the oxygen enrichment, which range from −4.74‰ to 1.02‰ (SD < 0.59‰), as listed in Table 3. A linear relationship (R^2^ = 0.94) seems to exist (as far as this can be assumed on the basis of three data points) between the δ^18^O values of ETN samples NS1, NS2 and NS3 (synthesized with the same erythritol sample ‘Sukrin 1019’), and the δ^18^O values of the nitrate salt precursors (Fig. S3 of the supplementary information). More samples should be added to confidently confirm this correlation.

### 3.5 Chemical attribution and forensic use

In this section two important applications of isotope analysis are discussed that could be beneficial in forensic explosives casework to answer profiling and intelligence questions related to the explosive ETN and its precursors. These applications are the forensic comparison of ETN samples and the linkage of ETN samples to its precursor materials.

#### 3.5.1 Sample comparison

Comparison of ETN samples to determine whether they could have originated from a similar source or synthetic batch is the most straightforward use of stable isotope analysis. For example, this could lead to evidence supporting the hypothesis that ETN explosive found in an intact IED at a crime scene is related to ETN material found in another IED or originating from a batch of ETN at the home of a suspect. An isotope ‘match’ between the two ETN samples, which is defined as a difference in isotope ratio that is within the measurement error, provides support for the hypothesis that the ETN samples originate from the same source. Whereas a ‘no-match’ between the isotopic values of two ETN samples typically excludes the same source hypothesis (as long as the observed differences cannot reasonably be explained). The evidential value of a ‘match/no-match’ given a certain hypothesis is determined by the characteristic nature of the observed isotope ratios, which depends on the frequency distributions of isotopic values present in ETN samples. The power to discriminate between ETN samples therefore depends on the isotopic ranges and distributions observed in a representative sample set, and the precision of the analysis. The consistent fractionation as demonstrated by the IRMS results in section 3.4 illustrate that the discrimination power is expected to ‘translate’ from the raw materials to the end product ETN. However, no meaningful DP analysis could be performed on the ETN data presented in this study because of the specific selection of raw materials to investigate isotope fractionation and precursor-end product linkage.

An essential condition which is required to use isotope ratios for sample differentiation, is the stability of the isotopic composition in the materials. In this study, stable carbon isotope compositions were observed between ‘fresh’ and ‘two years old’ ETN samples synthesized using the same erythritol precursors under similar reaction conditions. This indicates that aging of ETN did not affect the isotopic composition of the samples, which allows comparison between intact recrystallized ETN samples independent of time of production. This is very important in real-case forensic scenarios where the moment of synthesis of the discovered or confiscated explosive material is almost always unknown, and where the storage conditions could also be unclear. Some of the erythritol precursors were also (at least) two years old during synthesis of the ‘fresh’ ETN. Therefore, the findings also illustrate the stability of the carbon isotopic composition of the erythritol precursor materials. This is very useful for erythritol source determinations, because ETN batches produced with the same erythritol source (under similar reaction conditions) will have the same carbon isotope ratio. However, this also implies that ETN batches synthesized at different moments, using the same erythritol and conditions, cannot be distinguished due to the stability of carbon isotopes in the precursor.

The opposite was observed for the stability of nitric acid. For this raw material the nitrogen isotope ratio slightly increased over time from the moment the bottle is opened. Therefore, ETN samples that are synthesized with a similar bottle of nitric acid might show slightly different nitrogen isotopic compositions depending on the age of the acid. As a result, ETN batches synthesized with the same nitric acid can be potentially differentiated based on their nitrogen isotope value when synthesized at different moments in time. This can both provide and limit options in a forensic investigation depending on the context of the case. Some caution is needed when making statements on source origin, as slight differences in δ^15^N values might be observed when ETN batches are synthesized with the same nitric acid precursor (up to 2‰ difference observed in this study over a period of two years).

No differences were observed in this study between ETN crystals and corresponding melt-cast samples. This useful characteristic of ETN allows for comparison between processed and unprocessed materials, as the isotopic signature is conserved. For example, this enables common source investigations in scenarios where melt-cast ETN is found in an intact IED at a crime scene and residual ETN crystals are found at a suspect’s clandestine production facility.

The isotopic distributions in the large set of erythritol precursors showed that it is possible for two different sources of erythritol to have the same isotopic composition. For example, the erythritol samples ‘Steviala Ery-pure 1220’ (δ^13^C = −13.45‰, δ^2^H = 4.00‰, δ^18^O = 28.88‰) and ‘Sukrin 1019’ (δ^13^C = −13.41‰, δ^2^H = 4.03‰, δ^18^O = 29.09‰) cannot be distinguished based on their multi-isotopic compositions. This could lead to a false positive ‘match’, *i.e.,* evidence supporting the common origin hypothesis, whereas ETN samples are actually prepared with two different erythritol precursors. The evidence correctly supports the same source hypothesis, but the ground truth is different since we are dealing with a random match. In these cases it might be beneficial to combine isotope analysis with the previously reported partially nitrated erythritol impurity analysis. Although the impurity analysis did not provide source information on the precursors used, the characteristic impurity profiles of the ETN samples could provide extra differentiation power to distinguish between ETN samples. Combining data from different techniques is expected to increase the robustness of the forensic comparison especially if the additional information provided is orthogonal (*i.e*. uncorrelated). Unfortunately, we were unable to investigate this in our studies because of the different time frame and focus in the sample ETN preparation.

#### 3.5.2 Linkage of ETN samples to precursors

**Fig. 8:**
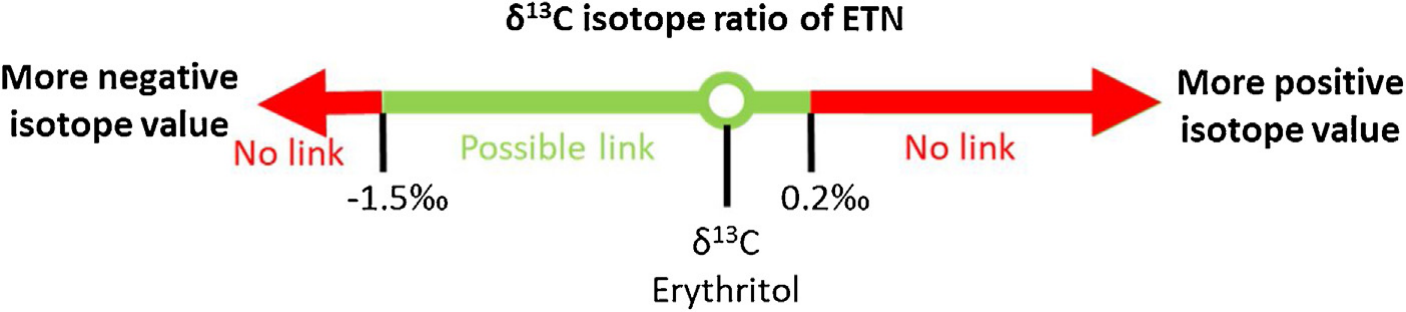
Visualization of the scenarios for potential linkage of ETN samples to its erythritol source based on the carbon isotope ratios observed in both materials.

The huge benefit of including isotope analysis in the framework of chemical attribution is the possibility to investigate which precursor materials could have been used to synthesize a specific batch of ETN explosive. The robust linear relationships between the carbon isotope ratios of the erythritol-ETN combinations allows linkage investigations in forensic scenarios where ETN is found in an intact IED at a crime scene and residues of erythritol are found at a home-laboratory. Fig. 8 shows the different scenarios for linkage related to the measured carbon isotope ratios of the evidence materials. If the ETN explosive material has a more positive δ^13^C value related to the erythritol material, the erythritol sample can most likely be excluded as precursor. In some cases, the non-erythritol components in the erythritol mixtures can result in a slightly higher δ^13^C value in the ETN samples. To investigate the influence of these non-erythritol components in more detail it might be beneficial to perform compound-specific IRMS that enables individual measurement of the erythritol part of the mixture. However, a more significant influence than observed in this study is unlikely, since the non-erythritol components only comprise a small amount of the mixture. Therefore, the positive cut-off value for exclusion was set at 0.2‰ positive carbon enrichment. Negative enrichments down to −1.5‰ were observed. Therefore, ETN material that has a δ^13^C value which is −1.5‰ more negative related to the erythritol material under investigation cannot have been synthesized with that erythritol source. More negative isotope values might be observed in ETN samples synthesized at very low reaction yields. However, the reaction yields in this study already ranged from 77.4% down to 14.2%, and it is unlikely that even lower yields are encountered in forensic cases. Perpetrators typically attempt to maximize the yield, and at yields below 15% raw material spillage and synthesis waste become excessive. The green line in Fig. 8, ranging from −1.5‰ up to 0.2‰ carbon isotopic enrichment (of ETN *versus* erythritol), shows the range that supports the hypothesis that the considered erythritol material was used as a precursor in the synthesis of ETN.

**Fig. 9:**
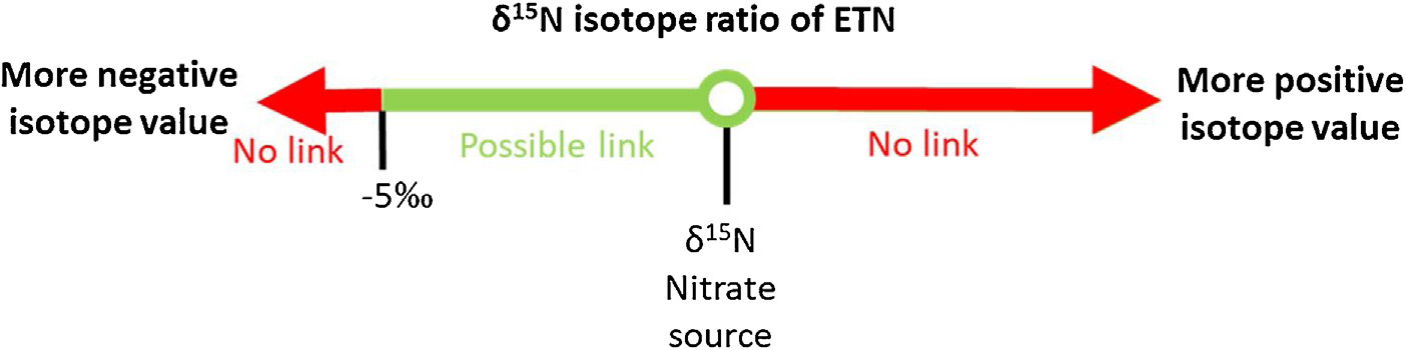
Visualization of the scenarios for potential linkage of ETN samples to its nitrate source based on the nitrogen isotope ratios observed in both materials.

Similar as for carbon, a robust linear relationship is observed for the nitrogen isotope values of the nitrate source-ETN combinations. The different scenarios for linkage of ETN to its nitrate precursor based on the nitrogen isotope values are depicted in Fig. 9. Negative enrichments are observed for all nitrate source-ETN combinations. This excludes a nitrate source as precursor for an ETN sample when the δ^15^N value of the ETN sample is more positive than the δ^15^N value of the suspected nitrate source. Negative nitrogen isotope enrichments down to −5‰ are observed when using ‘fresh’ nitric acid. The nitrogen isotopic composition of ‘aged’ nitric acid becomes heavier, leading to less negative δ^15^N values in the ETN product. Therefore, the negative enrichment of −5‰ can be regarded as the maximum isotopic shift and this excludes a nitrate source-ETN link when there is a larger negative shift. Again, the green line in Fig. 9, ranging from 0‰ down to −5‰ nitrogen enrichment shows the scenario that supports the hypothesis that the nitrate source material under investigation was used to synthesize the ETN explosive.

For hydrogen and oxygen isotope ratios, the relationship between the ETN product and its precursors is more complex. This makes it difficult to use these isotopes to support or refute hypotheses of linkage between ETN-precursor combinations. However, in some scenarios hydrogen and oxygen isotopic values can be used to support the hypothesis of exclusion. In all ETN samples prepared in this study, large negative isotopic shifts were observed related to the δ^2^H value of the erythritol precursor. Therefore, an erythritol material can be excluded as a source when the ETN sample has a more positive hydrogen isotope ratio. Although the magnitude of the negative isotopic shift is smaller for the δ^18^O values of the ETN-erythritol combinations, the same principle holds for the oxygen isotopic shifts and erythritol sources can be excluded when the δ^18^O values are more negative than the ETN sample under investigation.

## 4 Conclusions

In this study IRMS analysis was used to differentiate between ETN samples synthesized with various precursor materials. The carbon (δ^13^C), nitrogen (δ^15^N), hydrogen (δ^2^H) and oxygen (δ^18^O) isotope ratio data provided a high discrimination power between precursors and ETN samples. The stability of ETN was demonstrated for ‘two-year old’ samples and ‘melt-cast’ samples. This enables comparison between ETN samples independent of age and appearance, which is very useful in forensic casework.

Robust linear relationships were observed between the carbon isotopes of erythritol-ETN combinations and the nitrogen isotopes of nitrate source-ETN combinations. This allows predictions of possible linkage between suspected precursor materials and ETN material in forensic explosives investigations. In addition, the large negative isotopic fractionations observed in ETN for hydrogen and oxygen can also assist source exclusion, *i.e.,* the δ^2^H and δ^18^O values of precursor materials used for synthesis are always more positive in comparison to the resulting ETN.

Especially, ‘non-matching’ isotopic profiles are very powerful to exclude suspected precursor materials as donor in ETN samples. In case of ‘matching’ isotopic profiles, the isotopic distributions and discrimination power of the precursors reported in this study can provide insight in the evidential value of such a ‘match’. Since erythritol is the exclusive donor of carbon atoms in ETN, the variation in carbon isotope ratios depends on the range observed in the erythritol sources. The wide variety of erythritol sources available in this study gives a good representation of the worldwide population. More variation might be observed in the future if erythritol consumption continues to grow. Similarly, the nitrogen range observed in the nitrate precursors, which is the exclusive donor of nitrogen atoms, determines the variation of nitrogen isotopic values in ETN. Compared to erythritol, the available nitrate salt sources were limited, but already in this limited sample set a large variation in nitrogen isotopic values was observed. More research is needed to study the worldwide variation of δ^15^N ratios in fuming nitric acid and nitrate salt sources. If the nitrogen isotopic range remains similar to the range observed in this study, this could provide options to distinguish between the mixed acid and nitrate salt synthesis route when δ^15^N values are found in ETN that are more in line with the nitrate salt values.

Overall, adding IRMS analysis to the framework of chemical attribution for forensic explosives intelligence is very useful to aid ETN sample comparison and identifying potential raw materials used for synthesis. In conditions where isotopic profiles are inconclusive, combining IRMS analysis with the previously reported LC–MS impurity analysis could result in additional differentiation options. In addition, IRMS and LC–MS data are intrinsically very complementary with isotope values providing information on which raw materials are used to prepare ETN, while the partially nitrated impurities can give insight on synthesis conditions. It should be stressed that the current methodology for ETN attribution is only suited for intact material. After an explosion the residues do not provide options for detailed raw material characterization due to the destructive and uncontrolled nature of the violent chemical reaction. To conclude, it is important to continue building and expanding frameworks for forensic chemical attribution and forensic intelligence, for instance, to provide powerful tactical leads after failed attempts to cause an explosion that could ultimately lead to elimination of an explosive threat.

## CRediT authorship contribution statement

**Karlijn Bezemer:** Conceptualization, Investigation, Writing - original draft, Visualization, Project administration. **Lindsay McLennan:** Investigation, Writing - review & editing, Visualization. **Rosanne Hessels:** Investigation, Writing - review & editing. **Jorien Schoorl:** Methodology, Investigation, Writing - review & editing. **Jos van den Elshout:** Investigation, Writing - review & editing. **Antoine van der Heijden:** Writing - review & editing, Supervision. **Annemieke Hulsbergen:** Conceptualization, Writing - review & editing. **Mattijs Koeberg:** Conceptualization, Writing - review & editing. **Taylor Busby:** Investigation, Writing - review & editing. **Alexander Yevdokimov:** Investigation, Writing - review & editing. **Eva de Rijke:** Conceptualization, Writing - review & editing, Supervision. **Peter Schoenmakers:** Conceptualization, Writing - review & editing, Supervision. **James Smith:** Writing - review & editing, Supervision. **Jimmie Oxley:** Conceptualization, Writing - review & editing, Supervision. **Arian van Asten:** Conceptualization, Methodology, Writing - review & editing, Supervision.
