# Supplementary material for: Chemical attribution of the homemade explosive ETN - Part II: Isotope ratio mass spectrometry analysis of ETN and its precursors
Source: Forensic Sci Int. 2020 Aug;313:None. doi: 10.1016/j.forsciint.2020.110344 (PMC10624562; doi:10.1016/j.forsciint.2020.110344)
Supplement: Supplementary file 1 [file mmc1.docx]

**Supplementary information**

**Chemical attribution of the homemade explosive ETN – Part II: Isotope ratio mass spectrometry analysis of ETN and its precursors**

**Additional tables and figures**

**Table S1.** Collection of erythritol precursors (39 samples in total) and their sample information on brand, composition, country and year of purchase, production (or distribution) country, batch number and expiration date.

**Table S2.** Collection of nitrate precursors (16 samples in total) and their sample information on brand, purchasing party, purity, lot number and production country.

**Table S3.** The carbon (δ^13^C), hydrogen (δ^2^H) and oxygen (δ^18^O) isotopic values of the erythritol precursors (39 samples in total, 2-5 repeat analyses (n) per sample). Isotopic values are expressed in ‰.

**Table S4.** The nitrogen (δ^15^N) and oxygen (δ^18^O) isotopic values of the nitrate precursors (16 samples in total, 3-9 repeat analyses (n) per sample).

**Table S5.** Overview of the melt-cast ETN (8 samples in total, 2-4 repeat analyses (n) per sample) prepared in 2019 from the mixed-acid ETN samples.

**Table S6.** Overview of the ETN sample set (28 samples in total, 2-5 repeat analyses (n) per sample) prepared in 2017 with varying synthesis conditions.

**Fig. S1.** Isotope values of precursors *versus* ETN product to study the correlation of δ^13^C values of erythritol *versus* ETN-NL 2017 and ETN 2019 samples (50 samples in total).

**Fig. S2.** Isotope values of precursors *versus* ETN product with ±2SD to study the correlation of δ^2^H values of erythritol *versus:* *(a)* ETN-MA 2019 samples (8 samples), *(b)* ETN-NS 2019 samples (7 samples), *(c)* ETN-NL-MA 2017 (11 samples), and *(d)* ETN-NL-NS samples (10 samples).

**Fig. S3.** Oxygen isotope values of nitrate precursors *versus* ETN product to study the correlation of ETN samples NS1, NS2 and NS3 synthesized with ‘Sukrin 1019’ erythritol precursor (R^2^ = 0.94).

**Table S1.** Collection of erythritol precursors (39 samples in total) and their sample information on brand, composition, country and year of purchase, production (or distribution) country, batch number and expiration date.

| **Sample ID** | **Erythritol brand** | **Composition** | **Purchased country and year** | **Production (distribution) country** | **Batch number** | **Expiration date** |
| --- | --- | --- | --- | --- | --- | --- |
| Steviala Ery-pure 1220 | Steviala Ery-pure | 100% Erythritol | NL 2017 | (Belgium) | - | 2020/12 |
| Sukrin 1019 | Sukrin | 100% Erythritol | NL 2017 | (Norway) | 22415 | 2019/10 |
| Greensweet 0918 | Greensweet | 100% Erythritol | NL 2017 | (Netherlands) | 4119 | 2018/09 |
| Konzelmanns 0120 | Konzelmann’s | 100% Erythritol | NL 2017 | (Germany) | 90005117 | 2020/01 |
| Real Foods | Real Foods | 100% Erythritol | US 2017 | USA | 2140626 | 2020/04 |
| Hoosier Hill | Hoosier Hill | 100% Erythritol | US 2017 | USA | 003542711-17021626 | 2020/02 |
| Xylitol USA | Xylitol USA | 100% Erythritol | US 2017 | USA | - | - |
| Acros | Acros | 100% Erythritol | US 2017 | Japan | A0350512 | - |
| Chem-Impex | Chem-Impex | 100% Erythritol | US 2017 | USA | ME056580701 | - |
| Greensweet 1120 (a) | Greensweet | 100% Erythritol | NL 2019 | (Netherlands) | 84164 | 2020/11 |
| Greensweet 1120 (b) | Greensweet | 100% Erythritol | NL 2019 | (Netherlands) | 84164 | 2020/11 |
| Mattison | Mattison | 100% Erythritol | NL 2019 | (Netherlands) | 41172002 | 2020/03 |
| Diet-Food | Diet-Food | 100% Erythritol | NL 2019 | China | - | 2020/09 |
| Sukrin 0820 | Sukrin | 100% Erythritol | NL 2019 | (Norway) | 1017308074 | 2020/08 |
| Steviala Ery-pure 0821 | Steviala Ery-pure | 100% Erythritol | NL 2019 | (Belgium) | - | 2021/08 |
| Konzelmanns 0820 | Konzelmann's | 100% Erythritol | NL 2019 | (Germany) | 90008518 | 2021/05 |
| ViVio | ViVio | 100% Erythritol | NL 2019 | China | 2010807-X | 2020/08 |
| Wiezucker | Wiezucker | 100% Erythritol | NL 2019 | (Germany) | ET0164 | 2020/07 |
| Vital Zukka | Vital Zukka | 100% Erythritol | Austria 2019 | (Austria) | - | 2021/01 |
| Sweetiva | Sweetiva | 100% Erythritol | Austria 2019 | (Austria) | 3408 | 2020/12 |
| Truvia | Truvia | 85% Erythritol | US 2017 | - | 153491 | 2013/12 |
| Sukrin Melis 0120 | Sukrin Melis | Erythritol with stevia | NL 2017 | (Norway) | 23176 | 2020/01 |
| Sukrin Plus 1217 | Sukrin plus | Erythritol with stevia | NL 2017 | (Norway) | 415254604 | 2017/12 |
| Sukrin Plus 0719 | Sukrin plus | Erythritol with stevia | NL 2019 | (Norway) | 816231368 | 2019/07 |
| Sukrin One | Sukrin:1 1:1 | Erythritol with stevia | NL 2019 | (Norway) | 102316 | 2021/11 |
| Steviala Kristal | Steviala Kristal Sweet | Erythritol with stevia | NL 2019 | (Belgium) | 87148 | 2020/11 |
| Sukrin Melis 0421 | Sukrin Melis | Erythritol with stevia | NL 2019 | (Norway) | 100945 | 2021/04 |
| Cviti | Nutrivalue Cviti | Erythritol with stevia | NL 2019 | India | scp-11 | 2018/01 |
| Natu Sweet | Natu Sweet Kristalle | Erythritol with stevia | Austria 2019 | (Austria) | 13010209 | 2021/05 |
| SteviOcal (a) | Naturally Sweet Stevi0cal | Erythritol with stevia | India 2019 | (India) | 12175003 | - |
| steviOcal (b) | Naturally Sweet Stevi0cal | Erythritol with stevia | India 2019 | (India) | 12175003 | - |
| Steviala Ery-bronze 1220 (a) | Steviala Ery-Bronze | Bronw sugar erythritol | NL 2017 | (Belgium) | - | 2020/12 |
| Sukrin Gold 0319 | Sukrin Gold | Brown sugar erythritol with stevia | NL 2017 | (Norway) | 23725 | 2019/03 |
| Sukrin Gold 0420 | Sukrin Gold | Brown sugar erythritol with stevia | NL 2019 | (Norway) | 100922 | 2020/04 |
| Steviala Ery-bronze 1220 (b) | Steviala Ery-Bronze | Brown sugar erythritol | NL 2019 | (Belgium) | - | 2020/12 |
| Candarel | Candarel Sugarly | Erythritol mix (‹0.5% other material) | NL 2017 | Chech Republic | L6284 | 2019/09 |
| Natural Mate | Natural Mate | Erythritol mix with sucralose | NL 2017 | (USA) | MSC0072 | 2019/12 |
| Swerve | Swerve | Erythritol mix with oligosaccharides | US 2017 | USA | 1703100A09 | 2019/01 |
| Florsod | EASIS Perfect Icing Florsod | Erythritol mix with other material | NL 2019 | (Danmark) | 101991 | 2021/10 |

**Table S2.** Collection of nitrate precursors (16 samples in total) and their sample information on brand, purchasing party, purity, lot number and production country.

| Sample ID | Brand | Purchased | Purity | Lot number | Production |
| --- | --- | --- | --- | --- | --- |
| KNO_3_ 1 | Fisher | TNO (NL) | - | 154053 | India |
| KNO_3_ 2 | Boom | TNO (NL) | - | - | Germany |
| KNO_3_ 3 | Fluka | TNO (NL) | ≥ 98.0% | 1418584V | - |
| KNO_3_ 4 | VWR | TNO (NL) | - | 12F220006 | - |
| KNO_3_ 5 | Acros | UvA (NL) | 99+% | A0277094 | USA |
| KNO_3_ 6 | Merck | UvA (NL) | - | A625463513 | Germany |
| KNO_3_ 7 | Sigma | UvA (NL) | ≥ 99.5% | BCBC7771V | Germany |
| KNO_3_ 8 | Aldrich | NFI (NL) | 99+% | 04303CC | Germany |
| KNO_3_ 9 | Aldrich | NFI (NL) | 99.999% | MKBQ1141 | USA |
| KNO_3_ 10 | Fisher | URI (USA) | - | 6D38207 | - |
| NH_4_NO_3_ | Sigma | TNO (NL) | - | - | - |
| NaNO_3_ | Acros | TNO (NL) | - | - | - |
| HNO_3_ 1 | Merck | TNO (NL) | 100% | Z0400150636 | - |
| HNO_3_ 2 | Sigma | URI (USA) | 100% | BCBR4722V | Germany |
| HNO_3_ 3 | Sigma | URI (USA) | 100% | BCBT4510 | Germany |
| HNO_3_ 4 | Sigma | URI (USA) | 100% | BCBT4510 | Germany |

**Table S3.** The carbon (δ^13^C), hydrogen (δ^2^H) and oxygen (δ^18^O) isotopic values of the erythritol precursors (39 samples in total, 2-5 repeat analyses (n) per sample). Isotopic values are expressed in ‰.

|  |  | *Carbon (n = 3)* | | *Hydrogen* | | | *Oxygen (n = 4)* | |
| --- | --- | --- | --- | --- | --- | --- | --- | --- |
| **Sample ID** | **Composition** | **δ^13^C** | **SD** | **δ^2^H** | **SD** | **n** | **δ^18^O** | **SD** |
| Steviala Ery-pure 1220 | 100% Erythritol | -13.45 | 0.03 | 4.00 | 1.30 | 5 | 28.88 | 0.14 |
| Sukrin 1019 | 100% Erythritol | -13.41 | 0.02 | 4.03 | 1.23 | 4 | 29.09 | 0.11 |
| Greensweet 0918 | 100% Erythritol | -12.09 | 0.03 | 4.96 | 0.56 | 5 | 29.84 | 0.48 |
| Konzelmanns 0120 | 100% Erythritol | -11.97 | 0.02 | 8.21 | 0.86 | 3 | 29.20 | 0.14 |
| Real Foods | 100% Erythritol | -11.82 | 0.02 | 10.53 | 0.95 | 3 | 27.55 | 0.15 |
| Hoosier Hill | 100% Erythritol | -11.72 | 0.05 | 18.48 | 0.30 | 4 | 26.87 | 0.11 |
| Xylitol USA | 100% Erythritol | -11.93 | 0.03 | 7.51 | 0.07 | 2 | 26.08 | 0.26 |
| Acros | 100% Erythritol | -12.68 | 0.04 | 28.25 | 0.05 | 2 | 26.03 | 0.04 |
| Chem-Impex | 100% Erythritol | -11.74 | 0.03 | 10.57 | 0.30 | 3 | 26.96 | 0.24 |
| Greensweet 1120 (a) | 100% Erythritol | -11.66 | 0.01 | 12.86 | 2.27 | 2 | 29.41 | 0.39 |
| Greensweet 1120 (b) | 100% Erythritol | -11.83 | 0.03 | 5.83 | 0.76 | 5 | 27.30 | 0.42 |
| Mattison | 100% Erythritol | -12.41 | 0.01 | 8.65 | 1.73 | 5 | 26.74 | 0.15 |
| Diet-Food | 100% Erythritol | -11.77 | 0.00 | 16.34 | 0.39 | 3 | 27.19 | 0.06 |
| Sukrin 0820 | 100% Erythritol | -12.13 | 0.02 | 6.00 | 0.88 | 5 | 28.67 | 0.16 |
| Steviala Ery-pure 0821 | 100% Erythritol | -13.59 | 0.00 | 13.67 | 0.73 | 5 | 28.98 | 0.11 |
| Konzelmanns 0820 | 100% Erythritol | -12.22 | 0.01 | 3.18 | 1.48 | 4 | 27.93 | 0.01 |
| ViVio | 100% Erythritol | -11.14 | 0.03 | 1.66 | 0.60 | 5 | 26.48 | 0.03 |
| Wiezucker | 100% Erythritol | -12.21 | 0.02 | 2.65 | 0.58 | 4 | 26.49 | 0.39 |
| Vital Zukka | 100% Erythritol | -13.79 | 0.03 | 14.75 | 3.56 | 2 | 27.67 | 0.09 |
| Sweetiva | 100% Erythritol | -14.10 | 0.01 | 9.85 | 0.77 | 3 | 27.12 | 0.15 |
| Truvia | 85% Erythritol | -11.98 | 0.01 | -9.25 | 0.99 | 4 | 24.34 | 0.22 |
| Sukrin Melis 0120 | Erythritol with stevia | -13.42 | 0.02 | 7.89 | 1.07 | 3 | 29.28 | 0.07 |
| Sukrin Plus 1217 | Erythritol with stevia | -13.79 | 0.02 | -0.31 | 1.46 | 4 | 28.56 | 0.29 |
| Sukrin Plus 0719 | Erythritol with stevia | -13.25 | 0.02 | 5.77 | 0.19 | 3 | 28.01 | 0.10 |
| Sukrin One | Erythritol with stevia | -12.27 | 0.03 | 4.33 | 0.69 | 5 | 28.55 | 0.28 |
| Steviala Kristal | Erythritol with stevia | -11.76 | 0.03 | 5.88 | 0.81 | 5 | 26.94 | 0.32 |
| Sukrin Melis 0421 | Erythritol with stevia | -13.78 | 0.07 | 8.09 | 0.16 | 4 | 27.68 | 0.69 |
| Cviti | Erythritol with stevia | -12.76 | 0.10 | 11.08 | 0.96 | 4 | 28.00 | 0.18 |
| Natu Sweet | Erythritol with stevia | -12.36 | 0.00 | 3.12 | 0.49 | 4 | 28.03 | 0.36 |
| SteviOcal (a) | Erythritol with stevia | -12.61 | 0.04 | 9.04 | 0.40 | 5 | 28.02 | 0.40 |
| steviOcal (b) | Erythritol with stevia | -12.78 | 0.02 | 12.13 | 0.75 | 5 | 28.16 | 0.19 |
| Steviala Ery-bronze 1220 (a) | Brown sugar erythritol | -18.46 | 0.15 | -17.19 | 1.48 | 4 | 28.52 | 0.20 |
| Sukrin Gold 0319 | Brown sugar erythritol with stevia | -14.10 | 0.02 | 3.67 | 1.09 | 3 | 29.09 | 0.36 |
| Sukrin Gold 0420 | Brown sugar erythritol with stevia | -13.86 | 0.01 | 9.07 | 0.82 | 2 | 27.69 | 0.62 |
| Steviala Ery-bronze 1220 (b) | Brown sugar erythritol | -13.75 | 0.03 | 14.30 | 0.91 | 3 | 28.97 | 0.12 |
| Candarel | Erythritol mix (‹0.5% other material) | -12.17 | 0.07 | 3.67 | 1.00 | 2 | 28.18 | 0.55 |
| Natural Mate | Erythritol mix with sucralose | -11.85 | 0.01 | 0.89 | 0.47 | 3 | 26.74 | 0.16 |
| Swerve | Erythritol mix with oligosaccharides | -14.01 | 0.35 | -2.15 | 1.91 | 3 | 27.93 | 0.12 |
| Florsod | Erythritol mix with other material | -12.35 | 0.05 | -1.86 | 0.57 | 4 | 27.06 | 0.25 |

**Table S4.** The nitrogen (δ^15^N) and oxygen (δ^18^O) isotopic values of the nitrate precursors (16 samples in total, 3-9 repeat analyses (n) per sample). Isotopic values are expressed in ‰.

|  |  | *Nitrogen* | | | *Oxygen (n = 5)* | |
| --- | --- | --- | --- | --- | --- | --- |
| **Sample ID** | **Brand** | **δ^15^N** | **SD** | **n** | **δ^18^O** | **SD** |
| KNO_3_ 1 | Fisher | 49.60 | 0.50 | 3 | 34.03 | 0.38 |
| KNO_3_ 2 | Boom | -1.17 | 0.08 | 3 | 27.75 | 0.42 |
| KNO_3_ 3 | Fluka | -33.25 | 0.40 | 3 | 21.81 | 0.38 |
| KNO_3_ 4 | VWR | -1.78 | 0.14 | 3 | 51.81 | 2.93 |
| KNO_3_ 5 | Acros | 58.82 | 1.25 | 3 | 32.60 | 0.42 |
| KNO_3_ 6 | Merck | 6.45 | 0.63 | 3 | 25.65 | 0.18 |
| KNO_3_ 7 | Sigma | 14.23 | 0.84 | 3 | 28.92 | 0.68 |
| KNO_3_ 8 | Aldrich | -0.09 | 0.74 | 3 | 25.53 | 2.19 |
| KNO_3_ 9 | Aldrich | -1.85 | 1.01 | 3 | 23.61 | 0.59 |
| KNO_3_ 10 | Fisher | -1.25 | 0.10 | 9 | - | - |
| NH_4_NO_3_ | Sigma | 2.09 | 0.02 | 3 | 25.59 | 0.37 |
| NaNO_3_ | Acros | -0.95 | 0.10 | 3 | 17.32 | 0.22 |
| HNO_3_ 1 | Merck | 5.23 | 0.08 | 3 | 25.57 | 0.37 |
| HNO_3_ 2 | Sigma | 4.73 | 0.13 | 9 | - | - |
| HNO_3_ 3 | Sigma | 5.99 | 0.10 | 8 | - | - |
| HNO_3_ 4 | Sigma | 5.96 | 0.08 | 6 | - | - |

**Table S5.** Overview of the melt-cast ETN samples (8 samples in total, 2-4 repeat analyses (n) per sample) prepared in 2019 from the mixed-acid ETN samples. Isotopic values are expressed in ‰.

|  | *Precursors* | | *Carbon (n = 3)* | | *Nitrogen (n = 3)* | | *Hydrogen* | | | *Oxygen (n = 4)* | |
| --- | --- | --- | --- | --- | --- | --- | --- | --- | --- | --- | --- |
| **Sample ID** | **Erythritol** | **Nitrate** | **δ^13^C** | **SD** | **δ^15^N** | **SD** | **δ^2^H** | **SD** | **n** | **δ^18^O** | **SD** |
| MC1 | Sukrin 1019 | HNO_3_ 1 | -13.59 | 0.01 | 2.57 | 0.01 | - | - | - | 22.39 | 0.21 |
| MC1a | Sukrin 1019 | HNO_3_ 1 | -13.66 | 0.00 | 2.22 | 0.02 | -6.16 | 2.20 | 4 | 22.11 | 0.5 |
| MC2 | Sukrin 0820 | HNO_3_ 1 | -12.43 | 0.04 | 2.31 | 0.01 | -20.41 | 0.02 | 2 | 22.27 | 0.14 |
| MC3 | Steviala Ery-bronze 1220(a) | HNO_3_ 1 | -18.62 | 0.08 | 2.27 | 0.04 | -38.25 | 3.63 | 3 | 21.98 | 0.55 |
| MC4 | Steviala Ery-bronze 1220(b) | HNO_3_ 1 | -13.98 | 0.03 | 2.30 | 0.02 | -18.41 | 3.90 | 2 | 21.91 | 0.09 |
| MC5 | Vivio | HNO_3_ 1 | -11.38 | 0.05 | 2.39 | 0.01 | - | - | - | 21.49 | 0.08 |
| MC6 | Acros | HNO_3_ 1 | -12.32 | 0.02 | 2.57 | 0.03 | -11.76 | 3.34 | 3 | 21.72 | 0.31 |
| MC7 | Real Foods | HNO_3_ 1 | -11.55 | 0.22 | 2.56 | 0.07 | -10.91 | 1.60 | 4 | 22.47 | 0.24 |

**Table S6.** Overview of the ETN samples (28 samples in total, 2-5 repeat analyses (n) per sample) prepared in 2017 with varying synthesis conditions. All ETN samples were prepared with the same ‘Sukrin 1019’ erythritol source. The nitrate source was sample HNO_3_ 1 (for the mixed acid route) and KNO_3_ 3 (for the nitrate salt route). The yields, isotope ratios and enrichment values of the ETN samples are listed. Isotopic values are expressed in ‰.

*Mixed acid (MA) route standard conditions:* Add 9.3mL of fuming nitric acid to erythritol/sulfuric acid at room temperature (RT), 1 hour at 35 °C and precipitate product in ice water.

*Nitrate salt (NS) route standard conditions:* Add erythritol to 10 g of nitrate salt in sulfuric acid at 15-20 °C, stir for 1 hour at RT and precipitate product in ice water.

|  |  |  | *Carbon^b^ (n = 3)* | | | *Nitrogen^b^ (n = 3)* | | |  |  |
| --- | --- | --- | --- | --- | --- | --- | --- | --- | --- | --- |
| **Sample ID** | **Synthesis conditions** | **Yield^a^** | **δ^13^C** | **SD** | **ε^c^** | **δ^15^N** | **SD** | **ε^d^** |  |  |
| NL-MA3A | Standard | 52.6 | -13.65 | 0.05 | -0.24 | 1.84 | 0.28 | -3.37 |  |  |
| NL-MA3B | Standard | 48.6 | -13.69 | 0.05 | -0.28 | 1.04 | 0.15 | -4.17 |  |  |
| NL-MA11 | Lower nitrate (4,65mL FNA) | 61.8 | -13.92 | 0.06 | -0.52 | 1.73 | 0.17 | -3.48 |  |  |
| NL-MA13 | Battery acid (sulfuric acid) | 49.0 | -13.78 | 0.02 | -0.38 | 0.99 | 0.07 | -4.22 |  |  |
| NL-MA14 | 5h reaction time | 55.2 | -13.59 | 0.04 | -0.18 | 2.08 | 0.05 | -3.13 |  |  |
| NL-MA19 | (NH4)2CO3 wash/recryst. | 44.6 | -13.50 | 0.03 | -0.09 | 1.52 | 0.14 | -3.69 |  |  |
| NL-MA20 | Ethanol recryst. | 39.2 | -13.57 | 0.08 | -0.16 | 1.25 | 0.22 | -3.96 |  |  |
| NL-MA21 | Methanol recryst. | 39.2 | -13.50 | 0.02 | -0.09 | 1.33 | 0.11 | -3.88 |  |  |
| NL-NS3A | Standard | 43.2 | -13.76 | 0.02 | -0.35 | -36.00 | 0.08 | -2.84 |  |  |
| NL-NS3B | Standard | 40.0 | -14.19 | 0.27 | -0.79 | -36.31 | 0.08 | -3.17 |  |  |
| NL-NS14 | Battery acid (sulfuric acid) | 26.0 | -14.47 | 0.34 | -1.07 | -36.15 | 0.16 | -3.00 |  |  |
| NL-NS19 | (NH4)2CO3 wash/recryst. | 47.6 | -13.78 | 0.02 | -0.38 | -35.88 | 0.02 | -2.72 |  |  |
| NL-NS20 | Ethanol recryst. | 18.4 | -13.80 | 0.04 | -0.40 | -35.65 | 0.10 | -2.48 |  |  |
| NL-NS21 | Methanol recryst. | 18.4 | -13.75 | 0.58 | -0.34 | -35.60 | 0.12 | -2.43 |  |  |
|  |  |  | *Hydrogen^b^* | | | *Oxygen^b^ (n = 4)* | | | | |
| **Sample ID** | **Synthesis conditions** | **Yield^a^** | **δ^2^H** | **SD (n)** | **ε^c^** | **δ^18^O** | **SD** | **ε^c^** | **ε^d^** |  |
| NL-MA3A | Standard | 52.6 | -11.98 | 1.04 (5) | -15.95 | 21.72 | 0.12 | -7.16 | -3.75 |  |
| NL-MA3B | Standard | 48.6 | -17.55 | 1.24 (3) | -21.49 | - | - | - | - |  |
| NL-MA11 | Lower nitrate (4,65mL FNA) | 61.8 | -15.94 | 0.00 (2) | -19.89 | 21.35 | 0.09 | -7.52 | -4.11 |  |
| NL-MA13 | Battery acid (sulfuric acid) | 49.0 | -12.90 | 0.44 (4) | -16.86 | 26.25 | 0.49 | -2.76 | 0.66 |  |
| NL-MA14 | 5h reaction time | 55.2 | -13.05 | 1.70 (4) | -17.01 | 22.30 | 0.34 | -6.60 | -3.19 |  |
| NL-MA19 | (NH4)2CO3 wash/recryst. | 44.6 | -17.27 | 1.09 (2) | -21.21 | 22.31 | 0.14 | -6.59 | -3.18 |  |
| NL-MA20 | Ethanol recryst. | 39.2 | -12.38 | 0.37 (3) | -16.34 | 22.35 | 0.11 | -6.55 | -3.14 |  |
| NL-MA21 | Methanol recryst. | 39.2 | -11.97 | 1.03 (5) | -15.94 | 22.56 | 0.11 | -6.35 | -2.93 |  |
| NL-NS3A | Standard | 43.2 | -15.42 | 0.23 (4) | -19.37 | 22.36 | 0.01 | -6.54 | 0.54 |  |
| NL-NS3B | Standard | 40.0 | -13.65 | 1.70 (4) | -17.61 | 21.64 | 0.12 | -7.24 | -0.17 |  |
| NL-NS14 | Battery acid (sulfuric acid) | 26.0 | -14.82 | 1.12 (5) | -18.77 | 29.33 | 0.28 | 0.23 | 7.36 |  |
| NL-NS19 | (NH4)2CO3 wash/recryst. | 47.6 | -13.18 | 2.84 (3) | -17.14 | 21.56 | 0.58 | -7.32 | -0.24 |  |
| NL-NS20 | Ethanol recryst. | 18.4 | -16.32 | 2.85 (4) | -20.27 | 23.10 | 0.05 | -5.82 | 1.26 |  |
| NL-NS21 | Methanol recryst. | 18.4 | -15.53 | 6.52 (5) | -19.48 | 22.40 | 0.08 | -6.50 | 0.58 |  |

^a^ Yields are expressed in %.

^b^ Isotopic values and enrichments (ε) are expressed in ‰.

^c^ ETN enrichment versus erythritol precursor.

^d^ ETN enrichment versus nitrate precursor.

**Fig. S1.** Isotope values of precursors *versus* ETN product to study the correlation of δ^13^C values of erythritol *versus* ETN-NL 2017 and ETN 2019 samples (50 samples in total).

Correlation formula: δ^13^C (ETN) = 0.9719 x δ^13^C (erythritol precursor) - 0.7294, with R^2^ = 0.97.

**Fig. S2.** Isotope values of precursors *versus* ETN product with ±2SD to study the correlation of δ^2^H values of erythritol *versus:* *(a)* ETN-MA 2019 (8 samples), *(b)* ETN-NS 2019 (7 samples), *(c)* ETN-NL-MA 2017 (11 samples), and *(d)* ETN-NL-NS (10 samples). The SD values have been determined based on three repeat analyses for carbon and at least two (2-5) repeat analyses for hydrogen.

**Fig. S3.** Oxygen isotope values of nitrate precursors *versus* ETN product to study the correlation of ETN samples NS1, NS2 and NS3 synthesized with ‘Sukrin 1019’ erythritol precursor (R^2^ = 0.94).
